# Dopamine promotes NMDA receptor hypofunction in the retina through D_1_ receptor-mediated Csk activation, Src inhibition and decrease of GluN2B phosphorylation

**DOI:** 10.1038/srep40912

**Published:** 2017-01-18

**Authors:** Renato Socodato, Felipe N. Santiago, Camila C. Portugal, Ivan Domith, Thaísa G. Encarnação, Erick C. Loiola, Ana L. M. Ventura, Marcelo Cossenza, João B. Relvas, Newton G. Castro, Roberto Paes-de-Carvalho

**Affiliations:** 1 Instituto de Investigação e Inovação em Saúde (i3S) and Instituto de Biologia Molecular e Celular (IBMC), Universidade do Porto, Porto, Portugal; 2 Program of Neurosciences, Fluminense Federal University, Niterói, Brazil; 3 Department of Neurobiology, Institute of Biology, Fluminense Federal University, Niterói, Brazil; 4 Department of Physiology and Pharmacology, Biomedical Institute, Fluminense Federal University, Niterói, Brazil; 5 Laboratory of Molecular Pharmacology, Institute of Biomedical Sciences, Rio de Janeiro Federal University, Rio de Janeiro, Brazil

## Abstract

Dopamine and glutamate are critical neurotransmitters involved in light-induced synaptic activity in the retina. In brain neurons, dopamine D_1_ receptors (D_1_Rs) and the cytosolic protein tyrosine kinase Src can, independently, modulate the behavior of NMDA-type glutamate receptors (NMDARs). Here we studied the interplay between D_1_Rs, Src and NMDARs in retinal neurons. We reveal that dopamine-mediated D_1_R stimulation provoked NMDAR hypofunction in retinal neurons by attenuating NMDA-gated currents, by preventing NMDA-elicited calcium mobilization and by decreasing the phosphorylation of NMDAR subunit GluN2B. This dopamine effect was dependent on upregulation of the canonical D_1_R/adenylyl cyclase/cAMP/PKA pathway, of PKA-induced activation of C-terminal Src kinase (Csk) and of Src inhibition. Accordingly, knocking down Csk or overexpressing a Csk phosphoresistant Src mutant abrogated the dopamine-induced NMDAR hypofunction. Overall, the interplay between dopamine and NMDAR hypofunction, through the D_1_R/Csk/Src/GluN2B pathway, might impact on light-regulated synaptic activity in retinal neurons.

Dopamine (DA) primes neural circuits implicated in motor behavior, cognition, neurodegeneration and vision^1^,^2^,^3^. Two classes of DA receptors mediate its actions: D_1_-like (D_1_ and D_5_) and D_2_-like (D_2_, D_3_ and D_4_), which are positively and negatively linked to adenylyl cyclase (AC), respectively. DA is present in the retina, where it modulates AC activity since early developmental stages^4^. DA also controls growth cone motility and neurite retraction via D_1_R in the developing retina^5^, suggesting that DA might be a morphogen for retinal neuronal progenitor cells. Moreover, Parkinson-diseased patients develop late visual impairment, possibly by changes in the responsiveness of retinal ganglion cells to DA^6^,^7^.

D_1_Rs have been shown to physically interact with NMDAR subunits in brain neurons^8^ and DA-triggered D_1_R activation is often associated with the potentiation of NMDAR channel activity in those cells^9^,^10^,^11^,^12^,^13^,^14^,^15^,^16^. NMDAR activity is implicated in the regulation of visual system development^17^,^18^, in retinal cell death^19^ and in light transduction^20^. On the other hand, NMDAR hypofunction is associated with psychiatric disorders^21^,^22^,^23^. Several metabotropic receptors modulate the activity and membrane trafficking of NMDARs by phosphorylating their large intracellular domains in a subunit-specific manner^24^. Interestingly, NMDARs may be more susceptible to direct regulation by non-receptor tyrosine kinases, such as Src and Fyn^25^,^26^, than by classical serine-threonine protein kinases like PKA and PKC^27^. Indeed, Src is required for NMDAR activity and NMDAR-dependent plasticity in the brain^28^,^29^,^30^,^31^,^32^.

Src belongs to the Src family of protein kinases (SFKs), which are a class of cytoplasmic tyrosine kinases highly conserved throughout metazoan evolution^33^. Activation of SFKs, including Src, depends on Tyr^416^ phosphorylation (in the activation loop) and Tyr^527^ dephosphorylation (in the C-terminal region)^33^,^34^,^35^,^36^,^37^. The ubiquitously expressed C-terminal Src kinase (Csk) is a major kinase regulating the phosphorylation of this C-terminal tyrosine^37^,^38^,^39^. In Csk knockout mice, a severe deficit in neural tube development leads to embryonic lethality, likely due to widespread overactivation of SFKs^40^. Likewise, Csk null cells, including retinal neurons^41^, display a dramatic increase in Src activity^42^. Furthermore, Csk, likely through the downregulation of SFKs activity, can inhibit the potentiation of NMDAR channel function in hippocampal synapses^43^. Therefore, to comprehend the signaling interplay between DA, Csk/Src and NMDARs might be of paramount importance for understanding activity-dependent plasticity of retinal circuitry under physiological and pathophysiological conditions.

Since D_1_Rs and Src can independently regulate NMDAR activity we hypothesized that D_1_Rs would control Src activity to regulate the functioning of NMDARs in retinal neurons. Here we reveal that exposing retinal neurons to DA triggers the activation of the D_1_R/cAMP/PKA/Csk pathway leading to Src inhibition. The inhibition of Src was responsible for decreasing the phosphorylation of NMDAR subunit GluN2B at Tyr^1472^, for reducing NMDAR-gated currents, and for preventing NMDA-evoked calcium mobilization, leading to NMDAR hypofunction. Overall, we unveiled a signaling pathway composed of PKA/Csk/Src/GluN2B that associates DA-induced D_1_Rs activation with NMDARs hypofunction in retinal neurons.

## Results

### D_1_Rs stimulation inhibits Src in neurites of retinal neurons

**Figure 1:**
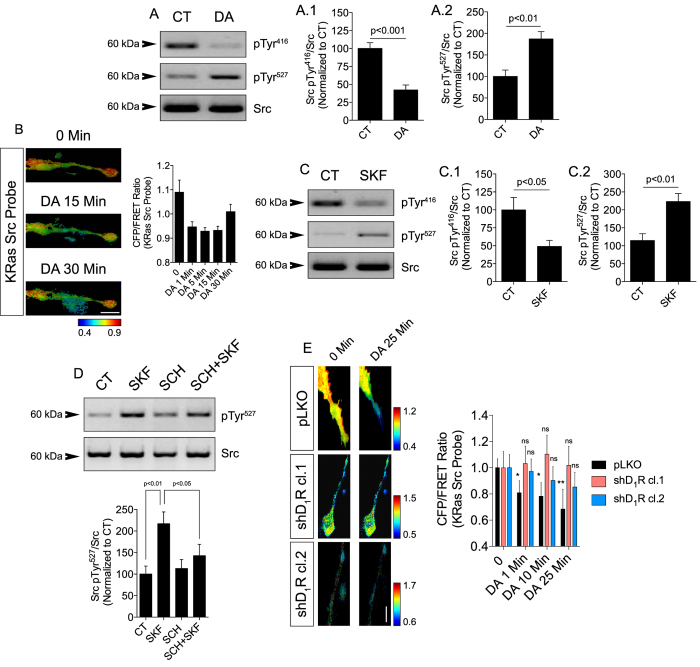
DA inhibits Src *via* activation of D_1_Rs. (**A**) Western blot for Src phosphorylated at Tyr^416^ (**A.1**) or Tyr^527^ (**A.2**) on lysates from retinal neuronal cultures treated for 30 min with DA (50 μM). Src was used as the loading control. Data are the mean ± SEM. N = 6 different and independent cultures, Student t test. (**B**) Retinal cultures expressing KRas Src FRET sensor were exposed to DA (10 μM). Time-lapse CFP/FRET ratios in neurites are coded as the pseudocolor ramp. Data are the mean ± SEM. N = 3 cells. Scale = 5 μm. (**C** and **D**) Western blotting against Src pTyr^416^ (**C.1** and **D**) or Src pTyr^527^ (**C.2**) on retinal cultures treated for 30 min with SKF-38393 (10 μM). In some cases neurons were pre-treated with SCH 23390 (10 μM). Data are the mean ± SEM. N = 6 different and independent cultures, Student t test (**C**) or One-way ANOVA (**D**). (**E**) Retinal neuronal cultures expressing pLKO (control; empty vector) or D_1_R shRNA were transfected with KRas Src FRET sensor and exposed to DA (10 μM; time = 0 min). Histograms (mean ± SEM) for pLKO (black) and shD_1_R (light red for shRNA clone 1 or light blue for shRNA clone 2) show time-lapse CFP/FRET ratio changes in the neurites. N = 3–4 neurons in each condition. *p < 0.05, **p < 0.01, Two-way ANOVA (related to time-point 0 in the pLKO group); ns = non-significant (related to time-point 0 in the shD_1_R groups). Scale = 5 μm. Uncropped Western blot gels related to this figure are displayed in Suppl. Fig. 7.

Activation of Src is dictated by the balance between the stimulatory phosphorylation of Tyr^416^ in its activation loop and the inhibitory phosphorylation of Tyr^527^ at its C-terminal region^38^. We first assessed the phosphorylation of Src at Tyr^416^ and Tyr^527^ residues by Western blotting in lysates from cultured retinal neurons (Fig. 1A). Stimulation of cultures with DA for 30 min induced a significant decrease in active Src (pTyr^416^; Fig. 1A.1) while it robustly increased inactive Src (pTyr^527^; Fig. 1A.2). To study the DA effect further we used a specific Src biosensor (KRas Src YPet^44^) and visualized by FRET-based time-lapse microscopy the subcellular activation of Src in neurites of living retinal neurons. We observed that DA treatment of retinal neurons expressing the Src FRET biosensor promoted fast and consistent inhibition of Src in neurites (Fig. 1B), indicating that DA decreases Src activation in retinal neurons.

Since D_1_Rs are expressed in the retina and DA can signal through these receptors in retinal neurons we therefore evaluated whether selective D_1_R modulation would also control the activation of Src. We observed by Western blotting that SKF-38393 (a selective D_1_R agonist) decreased active Src (pTyr^416^) and increased inactive Src (pTyr^527^) in retinal neurons (Fig. 1C.1 and 1C.2). In addition, the SKF-38393-induced Src pTyr^527^ increase was abolished by pre-treating neurons with the selective D_1_R antagonist SCH-23390 (Fig. 1D). We further confirmed the specific effect of D_1_R in inhibiting Src by FRET with the KRas Src biosensor in neurites of living retinal neurons knocked down for D_1_R (Fig. 1E light red bars; knockdown validated in Suppl. Fig. 1). To validate our *in vitro* findings in a more physiological model, we performed *ex vivo* experiments with DA and SKF-38393 in intact retinas acutely-isolated. Corroborating our culture data, DA or SKF-38393 increased Src pTyr^527^ density in the intact *ex vivo* retina (Suppl. Fig. 2).

### DA inhibits Src via the D_1_R canonical pathway in retinal neurons

**Figure 2:**
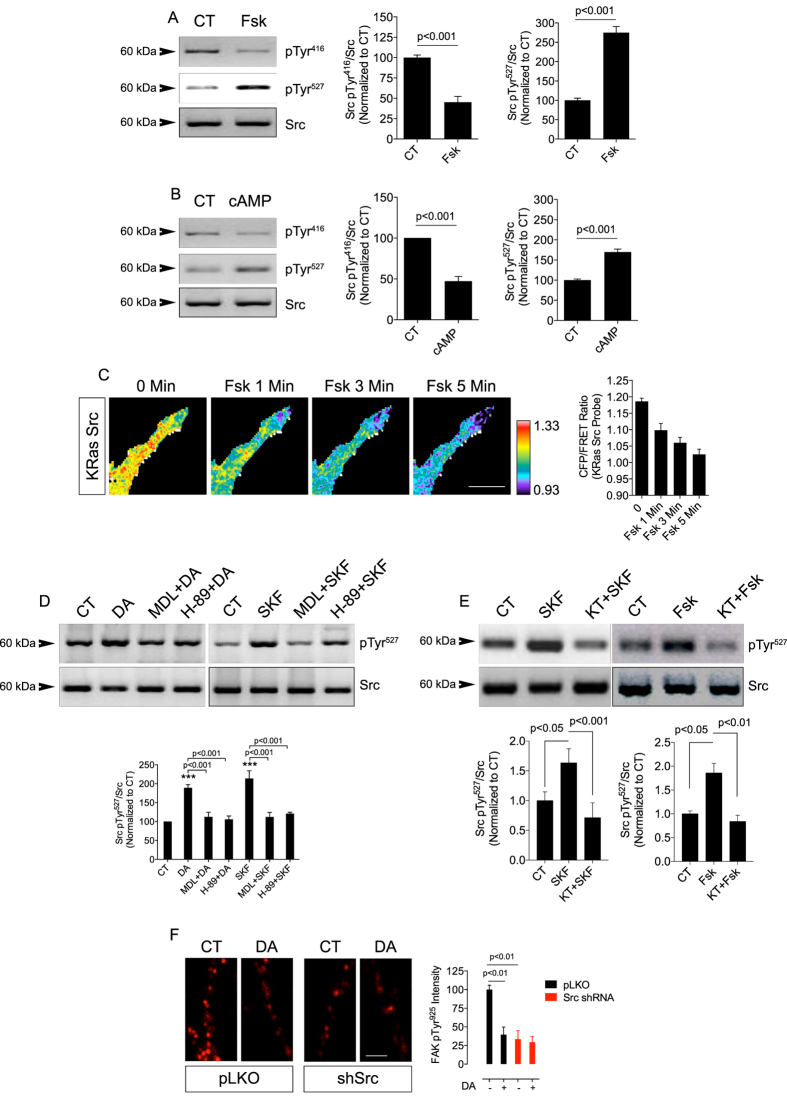
Canonical D_1_R pathway mediates Src inhibition. (**A** and **B**) Src phosphorylation at Tyr^416^ and Tyr^527^ mediated by forskolin (Fsk; 50 μM; (**A**) or 8Br-cAMP (100 μM; **B**) in retinal neuronal cultures. Src was used as the loading control. Data are the mean ± SEM. N = 3 different and independent cultures, Student t test. (**C)** Retinal neuronal cultures expressing KRas Src FRET sensor were exposed to Fsk (50 μM). Time-lapse CFP/FRET ratio changes in the neurites were normalized at 0 min and are coded as the pseudocolor ramp. Data are the mean ± SEM. N = 3 neurons. Scale = 5 μm. (**D**) Retinal neuronal cultures were pre-treated for 10 min with H-89 (15 μM) or MDL-12,330 A (10 μM) and then stimulated for 30 min with DA (50 μM) or SKF-38393 (10 μM). Src phosphorylation at Tyr^527^ was evaluated and Src served as the loading control. Data are the mean ± SEM. N = 3 different and independent cultures ***p<0.001 vs. CT (One-way ANOVA). (**E**) Retinal neuronal cultures were pre-treated for 10 min with KT-5720 (1 μM) and then stimulated for 30 min with SKF-38393 (10 μM) or Fsk (50 μM). Src phosphorylation at Tyr^527^ was evaluated and Src served as the loading control. Data are the mean ± SEM. N = 3 different and independent cultures, One-way ANOVA. (**F**) Confocal imaging of retinal neuronal cultures infected with lentiviruses carrying a control vector (pLKO; CT) or Src shRNAs and immunostained for FAK pTyr^925^ (red). DA (50 μM; 30 min). Data are the mean ± SEM. N = 3 different and independent cultures, One-way ANOVA. Uncropped Western blot gels related to this figure are displayed in Suppl. Fig. 8.

In most tissues, canonical D_1_R signaling occurs through adenylyl cyclase (AC), accumulation of cAMP and PKA activation^45^. We then asked whether this canonical D_1_R pathway would play a role in the DA-mediated Src inhibition. In this particular, forskolin (Fsk), which directly stimulates AC, or 8Br-cAMP, a permeable cAMP analog that directly activates PKA, were used. Both compounds decreased Src pTyr^416^ and increased Src pTyr^527^ in retinal cultures (Fig. 2A and B). Fsk also induced fast Src inhibition in neurites of living retinal neurons expressing the Src FRET biosensor (Fig. 2C). Blocking AC with MDL-12330A or PKA with H-89 prevented both the DA and the SKF-38393-induced Src pTyr^527^ increase (Fig. 2D). To corroborate the PKA effect on D_1_R-mediated Src inhibition we used another PKA inhibitor (KT-5720). Pre-incubation of retinal neurons with KT-5720 completely blocked the increase of Src pTyr^527^ elicited by the activation of D_1_Rs with SKF-38393 or by the activation of AC with Fsk (Fig. 2E). We further analyzed the modulation of Src function by DA using FAK pTyr^925^ as a functional index for Src activity^46^. As expected, DA as well as Src shRNA (validated in Suppl. Fig. 3A) decreased FAK pTyr^925^ levels and DA had no additional effect in cells treated with Src shRNA (Fig. 2F). Furthermore, Src shRNA-induced FAK pTyr^925^ decrease was not related with an increase in neuronal cell death (Suppl. Fig. 3B).

### Canonical D_1_R pathway activates Csk to inhibit Src in retinal neurons

**Figure 3:**
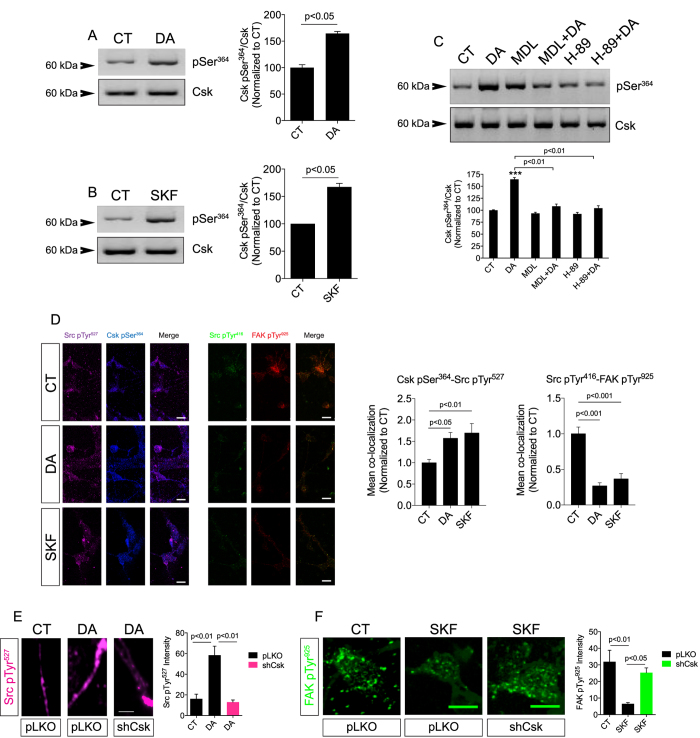
D_1_Rs activate Csk to inhibit Src. (**A** and **B**) Csk phosphorylation at Ser^364^ induced by DA (50 μM; 30 min; (**A**) or SKF-38393 (10 μM; 30 min; (**B**) in retinal neuronal cultures. Csk was used as the loading control. Data are the mean ± SEM. N = 3 different and independent cultures, Student t test. (**C**) Retinal neuronal cultures were pre-incubated for 10 min with H-89 (15 μM), MDL-12,330 A (10 μM) and then incubated with DA (50 μM; 30 min). Next, Western blot for Csk pSer^364^ was carried out. Csk was used as the loading control. Data are the mean ± SEM. N = 3 different and independent cultures. ***p < 0.001 vs. CT, One-way ANOVA. (**D**) Retinal neuronal cultures were stimulated with DA or SKF-38393 for 30 min, and then immunolabeled for Src pTyr^527^ (purple) and Csk pSer^364^ (blue) or Src pTyr^416^ (green) and FAK pTyr^925^ (red). Co-localization data are displayed as the mean ± SEM. N = 3 different and independent cultures, One-way ANOVA. Calibration bars = 10 μm. (**E** and **F**) Retinal neuronal cultures were infected with lentiviruses carrying Csk shRNAs and immunolabeled for Src pTyr^527^ (purple; **E**) or FAK pTyr^925^ (green; **F**) and stimulated with DA (50 μM; 30 min) or SKF-38393 (10 μM; 30 min). Data are the mean ± SEM. N = 3 different and independent cultures, One-way ANOVA. Calibration bars: (**E)** = 5 μm; (**F**) = 10 μm. Uncropped Western blot gels related to this figure are displayed in Suppl. Fig. 9.

We hypothesized that D_1_R-mediated PKA stimulation would regulate Csk activation since Csk is an endogenous Src repressor^42^. Moreover, PKA can stimulate Csk directly by phosphorylating the Ser^364^ at Csk kinase domain^47^. Indeed, DA or SKF-38393 increased Csk pSer^364^ in retinal neurons significantly (Fig. 3A and B) while inhibiting AC with MDL-12330A or blocking PKA with H-89 abrogated the DA effect (Fig. 3C). Confocal microscopy analysis showed that Src pTyr^527^ and Csk pSer^364^ were co-localized in puncta in retinal neurons and DA or SKF-38393 increased the Src pTyr^527^/Csk pSer^364^ co-localization puncta in most neurons (Fig. 3D, left panels), suggesting that Csk might regulate Src inhibition via D_1_R activation. DA and SKF-38393, as expected, decreased the Src pTyr^416^/FAK pTyr^925^ co-localization puncta (Fig. 3D, right panels).

To directly associate Csk with D_1_R-mediated Src inhibition, we knocked down Csk (validated in Suppl. Fig. 4) and evaluated whether D_1_Rs could still inhibit Src. Indeed, DA increased Src pTyr^527^ significantly in neurites of control neurons but not in neurites of neurons knocked down for Csk (Fig. 3E). In addition, when Csk was knocked down, the activation of D_1_Rs with SKF-38393 could not decrease FAK pTyr^925^ puncta (Fig. 3F), indicating that D_1_Rs inhibit Src via Csk activation. Data in acutely isolated retinas further showed that DA or SKF-38393 increased Csk phosphorylation via activation of D_1_Rs (Suppl. Fig. 5A), confirming that D_1_R activation of Csk inhibits Src in the retina.

### The D_1_R/Csk/Src pathway decreases GluN2B phosphorylation in retinal neurons

**Figure 4:**
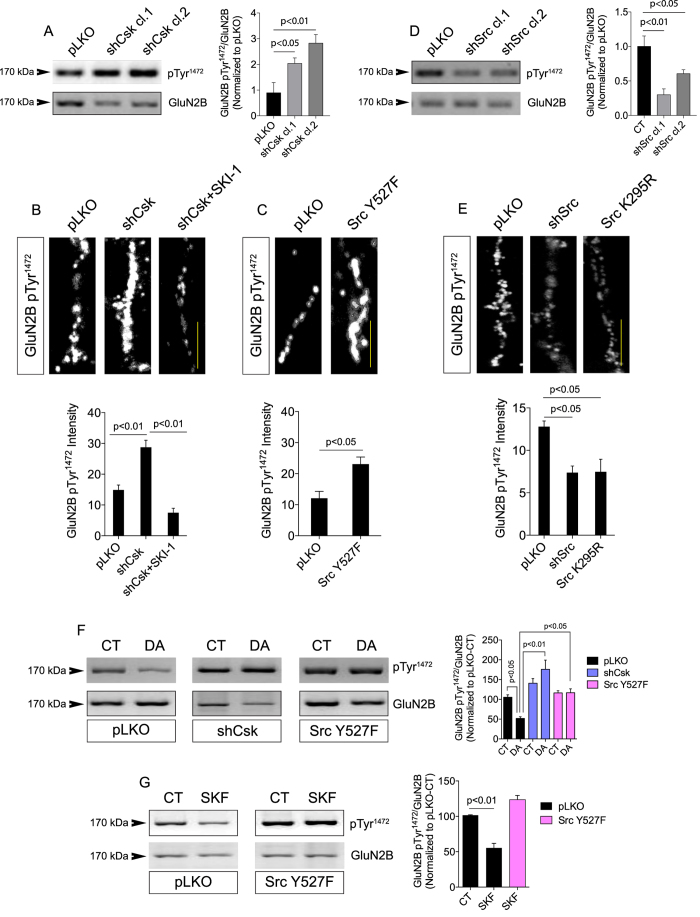
DA regulates GluN2B phosphorylation via Csk/Src pathway. (**A**) Representative Western blot for GluN2B pTyr^1472^ in extracts from retinal neuronal cultures expressing the empty vector pLKO or Csk shRNA (clone 1 or clone 2). GluN2B was used as the total. N = 3 different and independent cultures, One-way ANOVA. (**B**) Retinal neuronal cultures were infected with viruses carrying Csk shRNAs or the empty vector pLKO. In C3, cultures were treated with SKI-1 (100 nM; 24 h) and then immunostained for GluN2B pTyr^1472^. Data are the mean ± SEM. N = 3 different and independent cultures, One-way ANOVA. Scale = 5 μm. (**C**) Retinal neuronal cultures were infected with viruses carrying a constitutively active Src construct (Src Y527F) or the empty vector pLKO. Cultures were then immunostained for GluN2B pTyr^1472^. Data are the mean ± SEM. N = 3 different and independent cultures, Student t test. Scale = 5 μm. (**D**) Representative Western blot for GluN2B pTyr^1472^ in extracts from retinal neuronal cultures expressing the empty vector pLKO or Src shRNA (clone 1 or clone 2). GluN2B was used as the total. N = 3 different and independent cultures, One-way ANOVA. (**E**) Retinal neuronal cultures were infected with viruses carrying a kinase-dead Src construct (Src K295R), Src shRNA or the empty vector pLKO. Cultures were then immunostained for GluN2B pTyr^1472^. Data are the mean ± SEM. N = 3 different and independent cultures, One-way ANOVA. Scale = 5 μm. (**F** and **G**) Retinal neuronal cultures were transduced with the empty vector pLKO (**F** and **G**), Csk shRNA (**F**) or Src Y527F (**F** and **G**). Neurons were left untreated (CT) or treated for 30 min with DA (50 μM; (**D**) or SKF-38393 (10 μM; (**E**). Western blot for GluN2B pTyr^1472^ was then carried out. GluN2B was the loading control. Data are the mean ± SEM. N = 3 different and independent cultures, One-way ANOVA (**F** and **G**). Uncropped Western blot gels related to this figure are displayed in Suppl. Fig. 10.

SFKs, including Src, are claimed to modulate long-term potentiation and synaptic plasticity by stimulating the activity of NMDAR in the brain^26^. The presence different subunit composition in NMDARs determines the functional properties of the receptor^48^. In addition, phosphorylation of NMDARs subunits by different classes of cytosolic protein kinases further contributes to fine-tuning the channel behavior upon agonist binding^24^,^26^,^27^. For instance, phosphorylation of GluN2B at Tyr^1472^ modulates NMDAR currents and NMDAR-dependent synaptic plasticity^31^. We therefore hypothesized that D_1_R-induced Src inhibition could affect the functioning of GluN2B-containing NMDARs in retinal neurons. Western blotting and confocal imaging data showed that Csk knockdown increased GluN2B pTyr^1472^ (Fig. 4A and B) and the Src inhibitor SKI-1 prevented this effect in neurites (Fig. 4A). Overexpressing a Src mutant carrying a point mutation in the Csk phosphorylation site (Src Y527F), which renders Src constitutively active^49^, also enhanced GluN2B pTyr^1472^ in neurites (Fig. 4C). On the contrary, knocking down Src (Fig. 4D) or overexpressing a kinase-dead Src mutant (Src K295R) was sufficient to decrease GluN2B pTyr^1472^ in neurites (Fig. 4E). Here we concluded that Src activation is sufficient for phosphorylating GluN2B subunits in retinal neurons.

Since D_1_R activates Csk and inhibits Src in retinal neurons we evaluated whether exposing these cells to DA would affect GluN2B phosphorylation. DA treatment decreased GluN2B pTyr^1472^ in control neuronal cultures but not in cultures knocked down for Csk or in cultures overexpressing Src Y527F (Fig. 4F). Activation of D_1_Rs with SKF-38393 also reduced GluN2B pTyr^1472^ in control neuronal cultures, but not in cultures overexpressing the constitutively active Src mutant (Fig. 4G). In addition, we confirmed that activation of D_1_Rs by DA also decreased GluN2B phosphorylation at Tyr^1472^ in *ex vivo* intact retinas (Suppl. Fig. S5B).

### Dopamine triggers NMDAR hypofunction through a D_1_R/Csk/Src signaling pathway in retinal neurons

**Figure 5:**
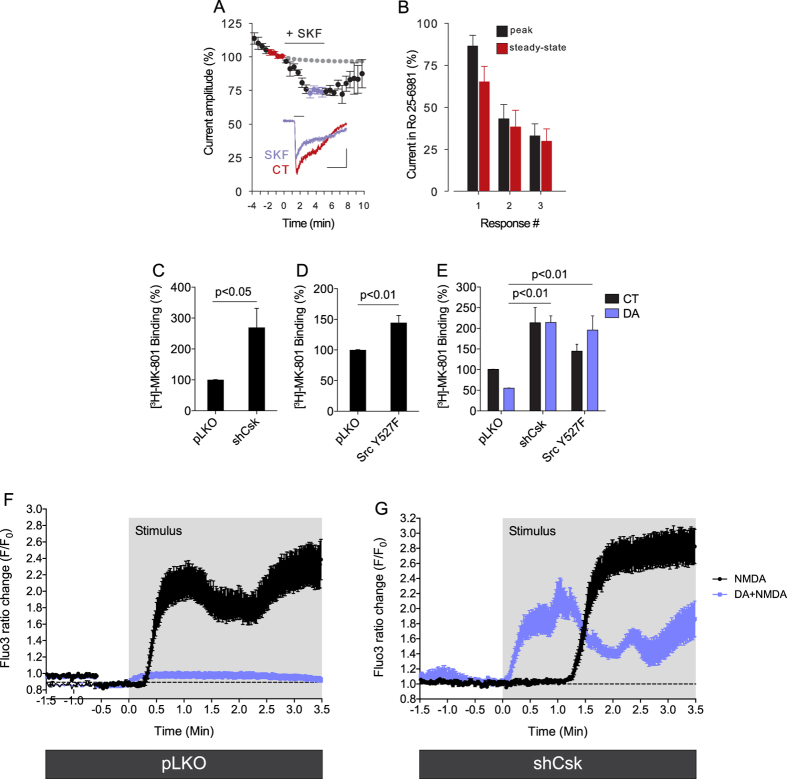
Activation of D_1_Rs reduces NMDAR-elicited responses in retinal neurons. (**A**) Current amplitudes expressed as % average of the last 4 CT responses before SKF-38393 5 μM (t = 0), N = 7 neurons. The gray dotted line indicates the predicted time course of spontaneous exponential decay of the responses, used as corrected control. The washout of SKF-38393 was followed in 3 of the 7 neurons. Inset: representative responses to NMDA (50 μM + glycine 5 μM; 1 s) in the absence and after 3–5 min in the presence of SKF-38393 (4 traces averaged; calibration: 2 s and 100 pA). (**B**) Ro 25–6981 2 μM was co-applied with NMDA + glycine at 1 min intervals after CT response. Peak and steady-state currents progressively reduced in the first three responses (N = 4 neurons). (**C–E)** Csk shRNA (**C** and **E**) or constitutively active Src (Src Y527F)-infected retinal neuronal cultures (**D** and **E**) were subjected to binding with radiolabeled MK-801. NMDARs activation was elicited by glutamate + glycine. Neurons were pre-treated with DA (50 μM; 30 min; (**E**). Data are the mean ± SEM. N = 3 different and independent cultures, Student t test (**C** and (**D**); Two-way ANOVA in (**E**). (**F** and **G**) Control (pLKO; (**F**) or Csk knocked down (shCsk; (**G**) retinal neuronal cultures were incubated with Fluo3 AM (5 μM; 1 h) and calcium imaging was performed in living retinal neurons. Cells were incubated with HBSS (saline; black circles) or with DA (10 μM; lilac circles) and recorded in a confocal microscope for 1.5 min (base line recording), followed by NMDA application (1 mM; 3.5 min; stimulus). Histograms are Fluo-3 F/F_0_ ratio changes ± SEM. N = 12 cells (each group in (**F**), 13 cells (**G)** NMDA) and 14 cells (**G**) DA + NMDA).

It is conceivable that the D_1_R-dependent decrease of GluN2B phosphorylation that we observed in retinal neurons (Fig. 4F and G) might affect NMDAR channel function. Therefore, to study NMDAR-dependent responses in retinal neurons, we first examined NMDA-gated currents in these cells. In nearly all neurons tested brief pulses of NMDA and glycine evoked whole-cell currents in the presence of 300 nM ZnCl_2_, which selectively inhibits GluN2A-containing receptors through a high-affinity site^50^,^51^,^52^. After achieving stable responses, activation of D_1_Rs by perfusing SKF-38393 reduced currents to 76.8 ± 2.6% of control (Fig. 5A). The SKF-38393-induced reduction in NMDAR-gated currents was consistent in each of 7 cells tested. Besides, in 2 of 3 cells tested, current amplitudes returned to control levels after a 5 min washout of SKF-38393 (Fig. 5A, grey circles). Co-application of NMDA with the selective GluN2B modulator Ro 25–6981 in long pulses (5–10 sec) led to partial use-dependent inhibition of current amplitudes (Fig. 5B), as expected for GluN1/GluN2B receptors^53^.

We then evaluated NMDAR functioning in large populations of retinal neurons using [^3^H] MK-801 binding assay in cultured neurons. Since [^3^H] MK-801 binding requires opened NMDAR channels at the neuronal plasma membrane^54^, cultures were pre-stimulated with glutamate and glycine. Knocking down Csk (Fig. 5C) or overexpressing the active Src mutant (Fig. 5D) increased glutamate-induced [^3^H] MK-801 specific binding when compared with control cultures, suggesting that Src activation increased glutamate-induced NMDAR functioning. Corroborating the electrophysiological results, DA decreased [^3^H] MK-801 specific binding in control cells whereas this effect was abrogated in cultures knocked down for Csk or in cultures overexpressing the Csk phosphoresistant Src mutant (Fig. 5E), indicating that DA-induced decrease of NMDAR functioning is mediated via Src inhibition.

Finally, we analyzed DA modulation of NMDA-elicited calcium mobilization. Calcium imaging in living retinal neurons revealed that pre-treatment with DA consistently abrogated NMDA-evoked calcium increase in these cells (Fig. 5F; lilac circles). This DA effect in retinal neurons depended on Src inhibition since the knockdown of Csk allowed a complete recovery of NMDA-evoked calcium increase in the presence of DA (Fig. 5G; lilac circles). Taken together, these data suggest that activation of D_1_Rs promotes NMDAR hypofunction via Csk activation and Src inhibition in retinal neurons.

## Discussion

The interaction between neurotransmitter systems is potentially important for understanding nervous system functioning and development, as well as neurodegenerative or neurodevelopmental disorders. Here we revealed a novel mechanism associating DA-dependent activation of D_1_R with NMDA receptor hypofunction in retinal neurons. We showed that activation of D_1_Rs, with consequent cAMP accumulation and PKA stimulation, promotes Csk activation, which in turn inhibits Src tyrosine kinase function. A direct consequence of Src inhibition is the decrease of the phosphorylation of GluN2B subunit at Tyr^1472^, leading to NMDAR hypofunction. Such signaling mechanism might correlate with the existence of a relay pathway composed of D_1_R, PKA, Csk, Src and GluN2B subunit that can efficiently depress NMDAR responses at glutamatergic synapses in retinal neurons.

Activation of GPCRs has been linked to Csk Ser^364^ phosphorylation^47^. In retinal neurons, cGMP-dependent kinase-mediated Src activation, controlled by calcium-permeable AMPA receptors and nitric oxide, does not involve Csk inhibition^41^. However, our data show that activation of D_1_Rs leads to Src inhibition via Csk activation in neurites of retinal neurons. In fibroblasts, GPCRs appear to enhance Csk activation through G protein βγ complexes but not via Gα_s_^55^. Herein, D_1_Rs promoted Csk activation in an AC/cAMP/PKA-dependent manner. The main effect of DA in Csk activation probably requires Gα_s_ mobilization, but Gβγ complexes might also participate in Csk recruitment to the plasma membrane upon DA activation of D_1_Rs. We did not evaluate the physical association of Csk and Src in steady state conditions or upon D_1_R activation in retinal neurons. However, preventing Csk phosphorylation, by pharmacological blockade of the cAMP/PKA pathway, or by depleting Csk, using shRNA-mediated Csk knockdown, abrogated the inhibition of Src induced by D_1_R activation, suggesting that there is a functional relationship between Csk and Src upon DA-mediated D_1_R modulation in retinal neurons.

Csk can decrease basal NMDAR activation by binding to GluN1 and GluN2 subunits and inactivating Src directly^43^. In line with this observation, we showed that Csk activation decreased GluN2B phosphorylation at Tyr^1472^ and inhibited NMDA-elicited calcium responses in living retinal neurons. In hippocampal neurons, inhibition of cAMP/PKA pathway by G_i/o_-coupled metabotropic glutamate 2/3 receptors reduces Csk activity and enhances the responses of GluN2A-containing NMDARs^56^. D_1_-like receptor activation and downstream regulation of PKA was also required for LTP induction in hippocampal slice preparations^57^, and for incrementing NMDAR functioning in the hippocampus^58^ or in the ventral tegmental area^59^. Our data, however, showed that D_1_R stimulation in retinal neurons promotes PKA activation leading to Csk-dependent Src inhibition, which then promotes the decrease of NMDAR responses. Taken together, these data suggest that NMDAR composed of GluN1/GluN2A or GluN1/GluN2B configurations might be subjected to bidirectional modulation by GPCRs activation, and downstream cAMP/PKA/Csk, in different neuronal populations. It will be of interest to clarify the functional consequences of cAMP/PKA/Csk modulation in GluN1/GluN2A/GluN2B heterotrimers, whose prevalence is high in the hippocampus but so far unknown in retinal neurons.

SFK-dependent phosphorylation of NMDAR subunits (for instance GluN2A or GluN2B) fine-tunes NMDAR gating^26^. Association of NADH dehydrogenase subunit 2 with Src unique domain mediates the functional coupling between Src and NMDARs^60^ and our data revealed that Src activation leads to the phosphorylation of GluN2B subunits and increased NMDAR functioning in retinal neurons. GluN2B subunit is expressed at synaptic sites in the retina^61^, suggesting an obvious role for GluN2B in regulating synaptic NMDAR activity in this tissue. By forcing Src activation (using shRNA to knockdown Csk expression or overexpressing a constitutively active Src mutant) we could prevent the D_1_R-mediated decrease of GluN2B phosphorylation and the DA-mediated NMDAR hypofunction in retinal neurons.

We ascertained that D_1_Rs indeed decreased NMDAR functioning in living retinal neurons by measuring NMDAR membrane currents and NMDA-evoked calcium mobilization. In this particular, the calcium imaging experiments showed that Csk was instrumental for the D_1_R-mediated decrease of NMDAR-elicited responses. The effect of DA in preventing NMDA-elicited cytosolic calcium increase was completely absent in neurons depleted of Csk, which corroborates that the DA effect on NMDA responses required Csk activation and Src inhibition, further suggesting that the D_1_R/Csk/Src pathway was responsible for the attenuation of NMDAR responses and not a direct channel blockade by DA^62^. Therefore, our results are in accordance with the existence of a signaling relay in retinal neurons, through which D_1_Rs, Csk, Src and NMDARs can regulate synaptic events in the retina.

In hippocampal neurons, NMDAR regulation by SFKs seems to be segregated: Fyn induces GluN2B phosphorylation while Src phosphorylates GluN2A^63^. In contrast, our data showed that direct activation of Src, by depleting Csk with shRNAs or by overexpressing a catalytically active Src mutant, in the absence of any stimulation of the D_1_R pathway was sufficient for promoting GluN2B phosphorylation in retinal neurons. C-terminal tyrosine phosphorylation of GluN2A or GluN2B by SFKs increases NMDAR channel function in different model systems^63^,^64^. Likewise, the signaling events leading to SFK-induced phosphorylation of NMDAR subunits, downstream of GPCRs activation, might be complex and also context-dependent in different neuronal populations. For instance, activation of the D_1_R/AC/PKA pathway in single CA1 neurons shows opposing effects on D_1_R-regulated NMDAR functioning, which is claimed to depend on NMDAR receptor localization and subunit composition^65^. Our observation that D_1_R activation was associated with a decrease of NMDAR-dependent currents in retinal neurons is consistent with Csk activation, Src inhibition and reduced GluN2B phosphorylation in neurites.

We revealed that activation of D_1_Rs by DA promptly upregulates the AC/cAMP/PKA cascade leading to PKA-dependent Csk Ser^364^ phosphorylation and activation, and Csk-induced Src Tyr^527^ phosphorylation in neurites. This Csk-induced phosphorylation renders Src inactive. Inactive Src, in turn, may not sustain the basal phosphorylation of GluN2B at Tyr^1472^, interfering with normal functioning of GluN2B-containg NMDARs, which culminates with NMDAR hypofunction in retinal neurons.

From the best of our knowledge, we unveiled a novel pathway linking DA-dependent D_1_R activation to a decrease in NMDAR functioning in retinal neurons. Since DA-mediated D_1_R modulation and glutamate-evoked NMDAR responses are pivotal for light transduction in the retinal tissue, we suggest that the association between D_1_Rs activation and NMDAR hypofunction, through the Csk/Src/GluN2B pathway, can fine-tune light-dependent neuronal activity in the retina.

### Experimental procedures

#### Study design

Our objective was to unveil the relationships between dopamine D_1_ receptor activation, the cytoplasmic protein tyrosine kinase Src and the functioning of the ionotropic glutamate receptor NMDA in retinal neurons. We used neuronal cultures obtained from developing retinal tissue from chicken, as well as intact retinas (acutely isolated) from embryonic day 11 chick embryos. We used different pharmacological compounds to modulate the activation of D_1_Rs (dopamine, SKF-38393 (selective D_1_ agonist) and SCH-23390 (selective D_1_ antagonist)), to regulate the canonical D_1_R pathway (cAMP, forskolin (direct activator of adenylyl cyclases), MDL-12,330 A (adenylyl cyclase blocker)) or to attenuate the activity of NMDARs (ZnCl_2_ (GluN2A inhibitor) or Ro 25–6981 (GluN2B blocker)). In addition, different expression vectors were employed to regulate the endogenous activity of Src in retinal neurons (lentiviruses-delivered shRNAs for knockdowns, overexpression of kinase dead or catalytically active mutants). Western blotting for detecting phospho-Src (active (Tyr^416^) or inhibited (Tyr^527^) on extracts from retinal cultures and FRET-based live cell imaging using a Src ratiometric nanosensor (KRas Src YPet chimera) determined Src activation/inhibition in retinal neurons. Western blotting and immunocytochemistry coupled to confocal microscopy assessed the phosphorylation of GluN2B subunit of NMDARs. Functional status of NMDAR in retinal neurons was assessed by a complement of 3 different methods: 1) electrophysiology (to study NMDAR gating); 2) radioligand biding in intact retinal neurons (using radiolabeled MK-801 to evaluate the functioning of fully operational NMDARs in a large population of retinal neurons); 3) NMDA-elicited calcium transients in living retinal neurons. Experimental units (cell cultures/intact retinas in Western blotting, immunocytochemistry and binding assays or retinal neurons in FRET experiments, electrophysiological recordings and calcium imaging) were randomly assigned from the different experimental groups (control, dopamine, SKF-38393, SCH, etc.) and then grouped accordingly. Investigators assessing and/or quantifying the results were blinded to the experimental groups and to the related experimental interventions (exposure to pharmacological agents and/or viral infections).

#### Reagents and Drugs

Dopamine; SKF-38393 (1-Phenyl -2,3,4,5-tetrahydro-(1 H)-3-benzazepine-7,8 -diol hydrochloride); SCH-23390 (7-Chloro-8-hydroxyl-3-methy-1-phenyl-2,3,4,5-tetrahydro -1H-3-benzazepine hydrochloride); MDL-12,330 A (cis-N-(2-Phenylcyclopentyl)- azacyclotridec-1-en-2-amine hydrochloride); H-89 (N-[2-(p-Bromocinnamylamino) ethyl]-5- isoquinolinesulfonamide dihydrochloride); Forskolin (7 β – Acetoxy - 8, 13 – epoxy – 1 α, 6 β,9α–trihydroxylabd–14–en–11–one); 8-Br-cAMP (8–Bromoadenosine 3′, 5′-cyclic monophosphate), BSA (Bovine serum albumin), Ro 25–6981, trypsin, MEM (minimum essential medium), Neurobasal, glutamate, glutamine, B27, gentamycin and FBS (fetal bovine serum) were from Thermo Scientific. Acrylamide, APS (ammonium persulphate), N,N′-Methylene-bisacrylamide, SDS (sodium dodecyl sulfate), TEMED (Tetramethyl-ethylenediamine), ECL kit, PVDF membranes and anti-rabbit HRP-conjugated secondary antibodies were from GE Healthcare. All other reagents were of analytical grade.

#### Antibodies

D_1_R antibody (ABN20; 1:100 or 1:50 for immunocytochemistry and 1:500 for Western blotting) was from Millipore. Monoclonal Src (clone 32G6; 1:2000), phospho-Src (Tyr^416^; 1:1000), phospho-Src (Tyr^527^; 1:100 for immunocytochemistry and 1:1000 for Western blotting), phospho FAK (Tyr^925^; 1:350) and monoclonal Csk (1:2000) antibodies were from Cell Signaling. Phospho-Csk (Ser^364^; 1:150 for immunocytochemistry and 1:1000 for Western blotting) and GluN2B (1:2500) were from Abcam. Phopho-GluN2B (Tyr^1472^; 1:100 for immunocytochemistry and 1:500 for Western blotting), alpha-Tubulin, Alexa Fluor^®^ 488, Alexa Fluor^®^ 568 and Alexa Fluor^®^ 594 were from Thermo Scientific. Primary antibodies were biochemically validated in Suppl. Fig. 6.

#### Plasmids

pLNCX chick Src Y527F (plasmid 13660), pLNCX chick Src K295R (plasmid 13659), pUMVC (Plasmid 8449), psPAX2 (plasmid 12260) and pMD2.G (plasmid 12259) were from Addgene. DRD1 Mission^®^ shRNA clones TRCN0000230251 (clone 1) and TRCN0000011335 (clone 2), Src Mission^®^ shRNA clones TRCN0000023597 (clone 1) and TRCN000023598 (clone 2), Csk Mission^®^ shRNA clones TRCN0000023735 (clone 1) and TRCN0000023736 (clone 2) were from Thermo Scientific.

#### Animals

Fertilized White Leghorn chicken eggs were obtained from a local hatchery and incubated at 38 °C and of 80–90% humidity. Procedures using chick embryos were all in accordance with the ‘Guide for the Care and Use of Laboratory Animals’ and were approved by the local commission of animal care CEPA/PROPPi from Federal Fluminense University, under the protocol 195/12. Efforts were made to minimize animal suffering and to reduce the number of animals used.

#### Retinal neuronal cultures

Retinas from eight-day-old chick embryos were dissected and digested in calcium and magnesium-free HBSS with 0.1% trypsin (w/v) for 17 min at 37 °C. Cells were suspended in minimum essential medium (supplemented with 3% FBS (v/v), 100 U/ml penicillin, 100 μg/ml streptomycin and 2 mM glutamine), dissociated using a glass pipette, and seeded onto 24- or 12-well culture plastic dishes in a density of 2 × 10^5^ cells/mm^2^ or onto live cell imaging plastic-bottom culture dishes (μ-Dish 35 mm, iBidi) at 1 × 10^5^ cells/mm^2^. Cells were maintained at 37 °C in a humidified incubator with 5% CO_2_, 95% air. Cellular composition and cell characterization in these cultures showed that 80–85% were neurons and 15–20% were glial cells^66^.

#### Lentiviruses production

Low passage HEK293T cells were seeded in 90 mm culture dishes. Cells with 80% confluence were co-transfected overnight with viruses-producing plasmids using Lipofectamine2000 (Thermo Scientific). Transfection ratios were as follows: 6 μg of shRNA plasmids to 3 μg of psPAX2 to 3 μg of VSVG (2:1:1). The next day, normal growth media replaced transfection media and cells were cultivated for an additional 48 h. Media with viral particles were collected, centrifuged at 1500 RPM for 5 min, and the supernatant was collected into new tubes.

#### Western blotting

For detection of the phosphorylation of indicated proteins, retinal cultures were washed in Hank’s balanced salt solution (HBSS), scraped off from culture dishes using 50–100 μl of RIPA buffer with protease inhibitor cocktail and the material was sonicated and protein content was determined by the BCA method. Samples were submitted to 9% *SDS-PAGE*, the proteins (45 μg/lane) were transferred to PVDF membranes which were next incubated overnight with primary antibodies. Subsequently, membranes were washed in TBS buffer (20 mM Tris; 200 mM NaCl), pH 7.6, incubated with peroxidase-conjugated secondary antibody and developed using an ECL chemiluminescence kit. Images were acquired in a ChemiDoc™ XRS+ System (BioRad), exported using Image Lab™ software (BioRad) and quantified by FIJI software.

#### Immunocytochemistry and confocal microscopy

Coverslips were fixed with 4% PFA, washed three times for 5 min in PBS, permeabilized with 0.1% Triton X-100 for 10 min, washed again and incubated for 1 h in blocking solution (5% BSA/3% FBS). Next, first primary antibody was added in blocking solution and coverslips were maintained in a humidified chamber for 1 h. Coverslips were washed three times for 10 min with PBS and incubated with the first secondary antibody for 1 h in blocking solution. Afterwards, blocking of immunoglobulin arms from the first secondary antibody was achieved by incubation with rabbit serum for 15 min followed by incubation with excess Fab anti-rabbit antibody fragment for 1 h. Then second primary antibody was incubated for 1 h in blocking solution, washed 5 times with PBS and second secondary antibody incubated for 1 h. Coverslips were washed three times for 10 min with PBS and mounted with Glycergel, visualized in a Leica SP5 II confocal microscope. Fluorescence intensity was determined using the LAS AF software (Leica Microsystems). Briefly, 16-bit images were acquired in sequential acquisition mode at a resolution of 1024 × 1024 pixels using identical gain and offset parameters. Pinhole was always kept at 1 airy. Values corresponding to pixels intensity in each group were exported using the LAS AF software and further evaluated in FIJI.

#### Quantification of fluorescent signals

Images were exported as raw 16-bit tiff using the LAS AF software with the original metadata preserved. Tiffs had their background subtracted in FIJI using the roller-ball ramp in between 10–50% pixel radius. Images of retinal neurites were segmented in FIJI using a panel of 12 different automatic local threshold algorithms for confocal images. Each thresholded neurite was delineated using the particle analyze tool in calibrated images and exported to FIJI ROI manager. Thresholded images were converted to binary mask using the dark background function. Binary mask images were multiplied for their respective original channel images using the image calculator plug-in to generate a masked 32-bit float images relative to each channel. Original coordinate vectors were retrieved from the ROI manager and FIJI returned the mean fluorescent intensity (MFI) in gray values contained within any single neurite using the multi-measure function. Individual MFI for single neurites were exported and statistically evaluated with the GraphPad Prism software.

#### Co-localization analysis

Images were acquired in Leica SP5 II confocal microscope using a HCX Plan Apo 63x/1.4–0.6 NA oil immersion objective in 16-bit sequential mode using bidirectional TCS mode at 100 Hz and the pinhole was kept at 1 airy. The LAS AF software processed images using thresholded background (+30% offset for both channels) and thresholded foreground (+13–15% offset for Csk pSer^364^ channel; +10–12% offset for Src pTyr^527^ channel; (+18–21% offset for Src pTyr^416^ channel; +12–18% offset for FAK pTyr^925^ channel). Values corresponding to Csk pSer^364^/Src pTyr^527^ or Src pTyr^416^/FAK pTyr^925^ pixel co-localization puncta in the neurites were retrieved from the LAS AF co-localization module, exported to Microsoft Excel and statistically evaluated by the GraphPad Prism software.

#### Cell death assessment

In the case of ethidium homodimer-1 (EthD1) labeling, cells were washed 2 x with HBSS and incubated with a solution of EthD1 (1:2000) in HBSS for 30 min. Then cells were fixed with 1% paraformaldehyde, coverslips were mounted in DAKO glycergel and visualized in a Leica TCS SP5 II confocal microscope. Cell viability was evaluated by counting the number of EthD1 positive cells in 6 different microscope fields per experimental group as previously described^67^. Experiments were always run in duplicate.

#### FRET-based live cell imaging and Src biosensor quantification

The excitation light source was a mercury metal halide bulb integrated with an EL6000 light attenuator. High-speed low vibration external filter wheels (equipped with CFP/YFP excitation and emission filters (Fast Filter Wheels, Leica Microsystems)) were mounted on a Leica DMI6000 B microscope. A 440–520 nm dichroic mirror (CG1, Leica Microsystems) and a PlanApo 63× 1.3NA glycerol immersion objective were used for CFP and FRET images. Images were acquired with 4 × 4 binning using a digital CMOS camera (ORCA-Flash4.0 V2, Hamamatsu Photonics). Shading illumination was online-corrected for CFP and FRET channels using a shading correction routine implemented for the LAS X software. At each time-point, CFP and FRET images were sequentially acquired using different filter combinations (CFP excitation plus CFP emission, and CFP excitation plus YFP emission, respectively).

Quantifications were performed as before^68^,^69^. In brief, acquired time lapses of living retinal neurons expressing the Src biosensor were exported as 16-bit tiff files and processed in FIJI software. Background was dynamically subtracted from all slices from both channels and images were filtered using a Kalman stack filter. Segmentation was achieved on a pixel-by-pixel basis using a modification of the Otsu algorithm. After background subtraction and thresholding, binary masks were generated for the CFP and FRET images. Original CFP and FRET images were masked, registered and bleach-corrected using a one-phase exponential decay function. Ratiometric images (CFP/FRET) for the Src biosensor (KRas Src YPet probe) were established as 32-bit float-point tiff images. Values corresponding to the mean gray values were generated using the multi calculation function in FIJI and exported as mentioned above.

#### Electrophysiology

Whole-cell patch-clamp recordings of NMDAR-gated currents were obtained and analyzed as previously described^62^, except that the membrane potential was held at −70 mV and fast NMDA pulses were applied with a U-tube perfusion system. Retinal cultures were continuously perfused with extracellular solution containing tetrodotoxin 0.15 μM and ZnCl_2_ 0.3 μM, which were also present in the solutions applied through the U-tube. One-second pulses of NMDA 50 μM and glycine 5 μM were applied every 30 s for 5–8 min, to establish a stable control response level after an initial spontaneous reduction (of 10–25%). Both the bath and U-tube solutions were then switched to observe the effect of added SKF-38393 5 μM. In some experiments, Ro 25–6981 2 μM was co-applied through the U-tube, in longer pulses. In this case, the currents both at the peak and at the steady state (mean value between 4 and 5 s of the pulse) were measured. Whole-cell capacitance was 5.0–15 pF and access resistance remained under 15 MΩ until the end of recordings.

#### [^3^H] MK-801 functional binding in intact retinal cells

In such paradigm, [^3^H] MK-801 binding only occurs by use-dependent NMDARs activation at the neuronal plasma membrane^70^. Total binding was measured in the presence of 5 nM [^3^H] MK-801 and non-specific binding was estimated in the presence of 5 nM [^3^H] MK-801 plus 50 μM of non-radioactive MK-801. Specific binding was defined calculating the difference between total and non-specific binding. All conditions were carried out in the presence of L-glutamate (50 μM) plus glycine (50 μM). Cultures were washed twice with HBSS without magnesium ions and then incubated with specified drugs for different time points in the absence or presence of unlabeled MK-801. At the last 5 minutes of the stimulation period, cultures were treated with 5 nM [^3^H] MK-801. The concentration used for glutamate and glycine was effective in saturating binding and 5 min incubation with [^3^H] MK-801 was sufficient to reach equilibrium. For the measurement of binding, cultures were homogenized in 0.1 M NaOH for 10 min and the radioactivity was determined in a Tri-Carb 2810TR Liquid Scintillation Analyzer (PerkinElmer). % Specific binding was plotted as fmol/mg protein.

#### Calcium imaging in living retinal neurons

Fluo3 AM indicator was used following the manufacturer protocol (Molecular Probes). In brief, retinal neuronal cultures were incubated for 1 h in complete MEM containing 5 μM Fluo-3 AM, 0.2% (v/v) pluronic F-127, 0.5% (v/v) DMSO and 2.5 mM probenecid. Cells were washed with HBSS with probenecid and incubated for an additional 15 min to allow complete de-esterification of AM ester. Live cell imaging was carried out on a Leica TCS SP5 II confocal microscope, using a 488 laser line for excitation. Emitted fluorescence was recorded between 530–565 nm. Ionomycin (2 μM) and a solution of 20 mM EGTA plus 1% (v/v) Triton-X100 were added sequentially at the end of the experiments to estimate maximal (F_max_) and minimal (F_min_) fluorescence intensities, respectively. Cells were recorded for 6–7 min in confocal live imaging mode. Baseline recording was stabilized for 90 s and bath NMDA (1 mM) was applied for 3.5 min followed by ionomycin and then EGTA/Triton. Free intracellular calcium variation (F/F_0_) was calculated for at least 12 cells *per* experimental group using consecutive responses evoked by NMDA (F) divided by baseline value just prior to NMDA application (F_0_). Control experiments were carried out to ensure that NMDA concentration used did not promote neuronal death and EGTA/Triton solution quenched fluorescence without promoting cell lysis during data recording. NMDA stimulation was evaluated only in neurons that responded sequentially to all treatments (NMDA, ionomycin and EGTA) during the recording period.

#### Acute retina preparations and *ex vivo* experiments

E11 retinas were dissected free from other ocular tissues, including the retinal pigmented epithelium, in ice-cold CMF, reserved in HBSS at 4 °C and then equilibrated in HBSS for 15 min at 37 °C. Next, retinas were incubated with designated drugs at 37 °C. After the incubation period, retinas were washed twice with HBSS at 4 °C, lysed with 5% (w/v) TCA on ice and centrifuged (15,000 RPM for 15 min at 4 °C). The pellet was re-suspended in sample buffer and protein content was determined by the Bradford method.

#### Statistical analysis

Significance in samples was evaluated by Student’s t test, One- or Two-way analysis of variance (ANOVA) followed by appropriate post-tests using the GraphPad Prism 6.0 software. In all tests a 95% confidence interval was used and p < 0.05 was considered as significant difference between sampled groups.

## Additional Information

**How to cite this article**: Socodato, R. *et al*. Dopamine promotes NMDA receptor hypofunction in the retina through D_1_ receptor-mediated Csk activation, Src inhibition and decrease of GluN2B phosphorylation. *Sci. Rep.* **7**, 40912; doi: 10.1038/srep40912 (2017).

**Publisher's note:** Springer Nature remains neutral with regard to jurisdictional claims in published maps and institutional affiliations.

## Supplementary Material

Supplementary Figures

## References

[b1] Carlsson A. & Lindqvist M. Effect of Chlorpromazine or Haloperidol on Formation of 3‐Methoxytyramine and Normetanephrine in Mouse Brain. Acta Pharmacol Toxicol (Copenh) 20, 140–144 (1963). 14060771 10.1111/j.1600-0773.1963.tb01730.x

[b2] Jackson C. R. . Retinal dopamine mediates multiple dimensions of light-adapted vision. J Neurosci 32, 9359–9368, doi: 10.1523/JNEUROSCI.0711-12.2012 (2012). 22764243 PMC3400466

[b3] Snyder S. H. The dopamine hypothesis of schizophrenia: focus on the dopamine receptor. Am J Psychiatry 133, 197–202, doi: 10.1176/ajp.133.2.197 (1976). 1251927

[b4] De Mello F. G. The ontogeny of dopamine-dependent increase of adenosine 3′,5′-cyclic monophosphate in the chick retina. J Neurochem 31, 1049–1053 (1978). 212530 10.1111/j.1471-4159.1978.tb00146.x

[b5] Lankford K. L., de Mello F. G. & Klein W. L. D1-type dopamine receptors inhibit growth cone motility in cultured retina neurons: evidence that neurotransmitters act as morphogenic growth regulators in the developing central nervous system. Proc Natl Acad Sci USA 85, 4567 (1988). 3380807 10.1073/pnas.85.12.4567-a PMC280472

[b6] Bodis-Wollner I. Visual deficits related to dopamine deficiency in experimental animals and Parkinson’s disease patients. Trends Neurosci 13, 296–302 (1990). 1695407 10.1016/0166-2236(90)90113-o

[b7] Masson G., Mestre D. & Blin O. Dopaminergic modulation of visual sensitivity in man. Fundam Clin Pharmacol 7, 449–463 (1993). 8294083 10.1111/j.1472-8206.1993.tb01041.x

[b8] Cepeda C. & Levine M. S. Where do you think you are going? The NMDA-D1 receptor trap. Sci STKE 2006, pe20 (2006). 16670371 10.1126/stke.3332006pe20

[b9] Chen G., Greengard P. & Yan Z. Potentiation of NMDA receptor currents by dopamine D1 receptors in prefrontal cortex. Proc Natl Acad Sci USA 101, 2596–2600 (2004). 14983054 10.1073/pnas.0308618100 PMC356995

[b10] Flores-Hernandez J. . Dopamine enhancement of NMDA currents in dissociated medium-sized striatal neurons: role of D1 receptors and DARPP-32. Journal of neurophysiology 88, 3010–3020, doi: 10.1152/jn.00361.2002 (2002). 12466426

[b11] Hallett P. J., Spoelgen R., Hyman B. T., Standaert D. G. & Dunah A. W. Dopamine D1 activation potentiates striatal NMDA receptors by tyrosine phosphorylation-dependent subunit trafficking. J Neurosci 26, 4690–4700, doi: 10.1523/jneurosci.0792-06.2006 (2006). 16641250 PMC6674081

[b12] Harvey J. & Lacey M. G. A postsynaptic interaction between dopamine D1 and NMDA receptors promotes presynaptic inhibition in the rat nucleus accumbens via adenosine release. J Neurosci 17, 5271–5280 (1997). 9204911 10.1523/JNEUROSCI.17-14-05271.1997 PMC6793812

[b13] Jiao H. . Dopamine D(1) and D(3) receptors oppositely regulate NMDA- and cocaine-induced MAPK signaling via NMDA receptor phosphorylation. J Neurochem 103, 840–848, doi: 10.1111/j.1471-4159.2007.04840.x (2007). 17897358

[b14] Pickel V. M., Colago E. E., Mania I., Molosh A. I. & Rainnie D. G. Dopamine D1 receptors co-distribute with N-methyl-D-aspartic acid type-1 subunits and modulate synaptically-evoked N-methyl-D-aspartic acid currents in rat basolateral amygdala. Neuroscience 142, 671–690, doi: 10.1016/j.neuroscience.2006.06.059 (2006). 16905271

[b15] Sarantis K., Matsokis N. & Angelatou F. Synergistic interactions of dopamine D1 and glutamate NMDA receptors in rat hippocampus and prefrontal cortex: involvement of ERK1/2 signaling. Neuroscience 163, 1135–1145, doi: 10.1016/j.neuroscience.2009.07.056 (2009). 19647050

[b16] Schoffelmeer A. N. . Synergistically interacting dopamine D1 and NMDA receptors mediate nonvesicular transporter-dependent GABA release from rat striatal medium spiny neurons. J Neurosci 20, 3496–3503 (2000). 10777812 10.1523/JNEUROSCI.20-09-03496.2000 PMC6773108

[b17] Blankenship A. G. & Feller M. B. Mechanisms underlying spontaneous patterned activity in developing neural circuits. Nat Rev Neurosci 11, 18–29, doi: 10.1038/nrn2759 (2010). 19953103 PMC2902252

[b18] Wong R. O. Retinal waves and visual system development. Annu Rev Neurosci 22, 29–47, doi: 10.1146/annurev.neuro.22.1.29 (1999). 10202531

[b19] Lam T. T., Abler A. S., Kwong J. M. & Tso M. O. N-methyl-D-aspartate (NMDA)–induced apoptosis in rat retina. Invest Ophthalmol Vis Sci 40, 2391–2397 (1999). 10476807

[b20] Diamond J. S. & Copenhagen D. R. The contribution of NMDA and non-NMDA receptors to the light-evoked input-output characteristics of retinal ganglion cells. Neuron 11, 725–738 (1993). 8104431 10.1016/0896-6273(93)90082-3

[b21] Schwartz T. L., Sachdeva S. & Stahl S. M. Glutamate neurocircuitry: theoretical underpinnings in schizophrenia. Front Pharmacol 3, 195, doi: 10.3389/fphar.2012.00195 (2012). 23189055 PMC3505861

[b22] Snyder M. A. & Gao W.-J. NMDA hypofunction as a convergence point for progression and symptoms of schizophrenia. Front Cell Neurosci 7, 31 (2013). 23543703 10.3389/fncel.2013.00031 PMC3608949

[b23] Snyder M. A. & Gao W. J. NMDA hypofunction as a convergence point for progression and symptoms of schizophrenia. Front Cell Neurosci 7, 31, doi: 10.3389/fncel.2013.00031 (2013). 23543703 PMC3608949

[b24] Chen B. S. & Roche K. W. Regulation of NMDA receptors by phosphorylation. Neuropharmacology 53, 362–368, doi: 10.1016/j.neuropharm.2007.05.018 (2007). 17644144 PMC2001266

[b25] Trepanier C. H., Jackson M. F. & MacDonald J. F. Regulation of NMDA receptors by the tyrosine kinase Fyn. The FEBS journal 279, 12–19, doi: 10.1111/j.1742-4658.2011.08391.x (2012). 21985328

[b26] Salter M. W. & Kalia L. V. Src kinases: a hub for NMDA receptor regulation. Nat Rev Neurosci 5, 317–328, doi: 10.1038/nrn1368 (2004). 15034556

[b27] MacDonald J. F., Kotecha S. A., Lu W. Y. & Jackson M. F. Convergence of PKC-dependent kinase signal cascades on NMDA receptors. Current drug targets 2, 299–312 (2001). 11554554 10.2174/1389450013348452

[b28] MacDonald J. F., Jackson M. F. & Beazely M. A. G protein-coupled receptors control NMDARs and metaplasticity in the hippocampus. Biochimica et biophysica acta 1768, 941–951, doi: 10.1016/j.bbamem.2006.12.006 (2007). 17261268

[b29] Lu Y. M., Roder J. C., Davidow J. & Salter M. W. Src activation in the induction of long-term potentiation in CA1 hippocampal neurons. Science 279, 1363–1367 (1998). 9478899 10.1126/science.279.5355.1363

[b30] Pitcher G. M. . Schizophrenia susceptibility pathway neuregulin 1-ErbB4 suppresses Src upregulation of NMDA receptors. Nature medicine 17, 470–478, doi: 10.1038/nm.2315 (2011). PMC3264662 21441918

[b31] Yang K. . Metaplasticity gated through differential regulation of GluN2A versus GluN2B receptors by Src family kinases. Embo j 31, 805–816, doi: 10.1038/emboj.2011.453 (2012). 22187052 PMC3280552

[b32] Manzerra P. . Zinc induces a Src family kinase-mediated up-regulation of NMDA receptor activity and excitotoxicity. Proc Natl Acad Sci USA 98, 11055–11061, doi: 10.1073/pnas.191353598 (2001). 11572968 PMC58682

[b33] Roskoski R. Jr. Src protein-tyrosine kinase structure, mechanism, and small molecule inhibitors. Pharmacological research 94, 9–25, doi: 10.1016/j.phrs.2015.01.003 (2015). 25662515

[b34] Martin G. S. The hunting of the Src. Nat Rev Mol Cell Biol 2, 467–475, doi: 10.1038/35073094 (2001). 11389470

[b35] Engen J. R. . Structure and dynamic regulation of Src-family kinases. Cellular and molecular life sciences: CMLS 65, 3058–3073, doi: 10.1007/s00018-008-8122-2 (2008). 18563293 PMC9357288

[b36] Ingley E. Src family kinases: regulation of their activities, levels and identification of new pathways. Biochimica et biophysica acta 1784, 56–65, doi: 10.1016/j.bbapap.2007.08.012 (2008). 17905674

[b37] Okada M. Regulation of the SRC family kinases by Csk. International journal of biological sciences 8, 1385–1397, doi: 10.7150/ijbs.5141 (2012). 23139636 PMC3492796

[b38] Okada M., Nada S., Yamanashi Y., Yamamoto T. & Nakagawa H. CSK: a protein-tyrosine kinase involved in regulation of src family kinases. J Biol Chem 266, 24249–24252 (1991). 1722201

[b39] Chong Y. P., Mulhern T. D. & Cheng H. C. C-terminal Src kinase (CSK) and CSK-homologous kinase (CHK)–endogenous negative regulators of Src-family protein kinases. Growth factors (Chur, Switzerland) 23, 233–244, doi: 10.1080/08977190500178877 (2005). 16243715

[b40] Imamoto A. & Soriano P. Disruption of the csk gene, encoding a negative regulator of Src family tyrosine kinases, leads to neural tube defects and embryonic lethality in mice. Cell 73, 1117–1124 (1993). 7685657 10.1016/0092-8674(93)90641-3

[b41] Socodato R. . Calcium-permeable alpha-amino-3-hydroxy-5-methyl-4-isoxazolepropionic acid receptors trigger neuronal nitric-oxide synthase activation to promote nerve cell death in an Src kinase-dependent fashion. J Biol Chem 287, 38680–38694, doi: 10.1074/jbc.M112.353961 (2012). 22992730 PMC3493912

[b42] Nada S. . Constitutive activation of Src family kinases in mouse embryos that lack Csk. Cell 73, 1125–1135 (1993). 8513497 10.1016/0092-8674(93)90642-4

[b43] Xu J. . Control of excitatory synaptic transmission by C-terminal Src kinase. J Biol Chem 283, 17503–17514, doi: 10.1074/jbc.M800917200 (2008). 18445593 PMC2427324

[b44] Ouyang M., Sun J., Chien S. & Wang Y. Determination of hierarchical relationship of Src and Rac at subcellular locations with FRET biosensors. Proc Natl Acad Sci USA 105, 14353–14358 (2008). 18799748 10.1073/pnas.0807537105 PMC2567163

[b45] Greengard P. The neurobiology of slow synaptic transmission. Science 294, 1024–1030, doi: 10.1126/science.294.5544.1024 (2001). 11691979

[b46] Schlaepfer D. D., Hanks S. K., Hunter T. & van der Geer P. Integrin-mediated signal transduction linked to Ras pathway by GRB2 binding to focal adhesion kinase. Nature 372, 786–791 (1994). 7997267 10.1038/372786a0

[b47] Yaqub S. . Activation of C-terminal Src kinase (Csk) by phosphorylation at serine-364 depends on the Csk-Src homology 3 domain. Biochem J 372, 271–278 (2003). 12600271 10.1042/BJ20030021 PMC1223381

[b48] Paoletti P., Bellone C. & Zhou Q. NMDA receptor subunit diversity: impact on receptor properties, synaptic plasticity and disease. Nat Rev Neurosci 14, 383–400, doi: 10.1038/nrn3504 (2013). 23686171

[b49] Nada S., Okada M., MacAuley A., Cooper J. A. & Nakagawa H. Cloning of a complementary DNA for a protein-tyrosine kinase that specifically phosphorylates a negative regulatory site of p60c-src. Nature 351, 69–72, doi: 10.1038/351069a0 (1991). 1709258

[b50] Chen N., Moshaver A. & Raymond L. A. Differential sensitivity of recombinant N-methyl-D-aspartate receptor subtypes to zinc inhibition. Molecular pharmacology 51, 1015–1023 (1997). 9187268 10.1124/mol.51.6.1015

[b51] Williams K. Separating dual effects of zinc at recombinant N-methyl-D-aspartate receptors. Neuroscience letters 215, 9–12 (1996). 8880741 10.1016/s0304-3940(96)12924-4

[b52] Paoletti P., Ascher P. & Neyton J. High-affinity zinc inhibition of NMDA NR1-NR2A receptors. J Neurosci 17, 5711–5725 (1997). 9221770 10.1523/JNEUROSCI.17-15-05711.1997 PMC6573217

[b53] Fischer G. . Ro 25-6981, a highly potent and selective blocker of N-methyl-D-aspartate receptors containing the NR2B subunit. Characterization *in vitro*. The Journal of pharmacology and experimental therapeutics 283, 1285–1292 (1997). 9400004

[b54] Ransom R. W. & Stec N. L. Cooperative modulation of [3H]MK-801 binding to the N-methyl-D-aspartate receptor-ion channel complex by L-glutamate, glycine, and polyamines. J Neurochem 51, 830–836 (1988). 2457653 10.1111/j.1471-4159.1988.tb01818.x

[b55] Lowry W. E. . Csk, a critical link of g protein signals to actin cytoskeletal reorganization. Dev Cell 2, 733–744 (2002). 12062086 10.1016/s1534-5807(02)00175-2

[b56] Trepanier C., Lei G., Xie Y. F. & MacDonald J. F. Group II metabotropic glutamate receptors modify N-methyl-D-aspartate receptors via Src kinase. Sci Rep 3, 926, doi: 10.1038/srep00926 (2013). 23378895 PMC3558700

[b57] Stramiello M. & Wagner J. J. D1/5 receptor-mediated enhancement of LTP requires PKA, Src family kinases, and NR2B-containing NMDARs. Neuropharmacology 55, 871–877, doi: 10.1016/j.neuropharm.2008.06.053 (2008). 18644393 PMC2578828

[b58] Murphy J. A. . Phosphorylation of Ser1166 on GluN2B by PKA is critical to synaptic NMDA receptor function and Ca2+ signaling in spines. J Neurosci 34, 869–879, doi: 10.1523/jneurosci.4538-13.2014 (2014). 24431445 PMC3891964

[b59] Schilstrom B. . Cocaine enhances NMDA receptor-mediated currents in ventral tegmental area cells via dopamine D5 receptor-dependent redistribution of NMDA receptors. J Neurosci 26, 8549–8558, doi: 10.1523/jneurosci.5179-05.2006 (2006). 16914681 PMC6674361

[b60] Gingrich J. R. . Unique domain anchoring of Src to synaptic NMDA receptors via the mitochondrial protein NADH dehydrogenase subunit 2. Proc Natl Acad Sci USA 101, 6237–6242, doi: 10.1073/pnas.0401413101 (2004). 15069201 PMC395953

[b61] Pourcho R. G., Qin P. & Goebel D. J. Cellular and subcellular distribution of NMDA receptor subunit NR2B in the retina. J Comp Neurol 433, 75–85 (2001). 11283950 10.1002/cne.1126

[b62] Castro N. G., de Mello M. C. F., de Mello F. G. & Aracava Y. Direct inhibition of the *N*-methyl-d-aspartate receptor channel by dopamine and (+)-SKF38393. Br. J. Pharmacol. 126, 1847–1855, doi: 10.1038/sj.bjp.0702479 (1999). 10372829 PMC1565957

[b63] Yang K. . Metaplasticity gated through differential regulation of GluN2A versus GluN2B receptors by Src family kinases. EMBO J 31, 805–816 (2011). 22187052 10.1038/emboj.2011.453 PMC3280552

[b64] Yu X. M., Askalan R., Keil G. J. 2nd & Salter M. W. NMDA channel regulation by channel-associated protein tyrosine kinase Src. Science 275, 674–678 (1997). 9005855 10.1126/science.275.5300.674

[b65] Varela J. A., Hirsch S. J., Chapman D., Leverich L. S. & Greene R. W. D1/D5 modulation of synaptic NMDA receptor currents. J Neurosci 29, 3109–3119, doi: 10.1523/JNEUROSCI.4746-08.2009 (2009). 19279248 PMC2684496

[b66] Portugal C. C. . Nitric Oxide Modulates Sodium Vitamin C Transporter 2 (SVCT-2) Protein Expression via Protein Kinase G (PKG) and Nuclear Factor-kappaB (NF-kB). J Biol Chem 287, 3860–3872 (2012). 22041898 10.1074/jbc.M111.260166 PMC3281729

[b67] Socodato R. . The nitric oxide-cGKII system relays death and survival signals during embryonic retinal development via AKT-induced CREB1 activation. Cell death and differentiation 21, 915–928, doi: 10.1038/cdd.2014.11 (2014). 24531539 PMC4013510

[b68] Socodato R. . c-Src deactivation by the polyphenol 3-O-caffeoylquinic acid abrogates reactive oxygen species-mediated glutamate release from microglia and neuronal excitotoxicity. Free Radic Biol Med 79, 45–55, doi: 10.1016/j.freeradbiomed.2014.11.019 (2015). 25486178

[b69] Socodato R. . c‐Src function is necessary and sufficient for triggering microglial cell activation. Glia 63, 497–511 (2015). 25421817 10.1002/glia.22767

[b70] Foster A. C. & Wong E. H. The novel anticonvulsant MK-801 binds to the activated state of the N-methyl-D-aspartate receptor in rat brain. British journal of pharmacology 91, 403–409 (1987). 2886170 10.1111/j.1476-5381.1987.tb10295.x PMC1853511

