# Supplementary material for: Dopamine promotes NMDA receptor hypofunction in the retina through D1 receptor-mediated Csk activation, Src inhibition and decrease of GluN2B phosphorylation
Source: Sci Rep. 2017 Jan 18;7:40912. doi: 10.1038/srep40912 (PMC5241882; doi:10.1038/srep40912)

Dopamine promotes NMDA receptor hypofunction in the retina through D_1 receptor-mediated Csk activation, Src inhibition and decrease of GluN2B phosphorylation

[5,*]Renato Socodato, [1]Felipe N. Santiago, [5]Camila C. Portugal, [1]Ivan Domith, [1]Thaísa G. da Encarnação, [1]Erick C. Loiola, [1,3]Ana L. M. Ventura, [1,2]Marcelo Cossenza, [5]João B. Relvas, [4]Newton G. Castro and [1,3,*]Roberto Paes-de-Carvalho.

[1]Program of Neurosciences; [2]Department of Physiology and Pharmacology, Biomedical Institute; [3] Department of Neurobiology, Institute of Biology, Fluminense Federal University, Niterói, Brazil.

[4]Laboratory of Molecular Pharmacology, Institute of Biomedical Sciences, Rio de Janeiro Federal University, Rio de Janeiro, Brazil.

[5]Instituto de Investigação e Inovação em Saúde (i3S) and Instituto de Biologia Molecular e Celular (IBMC), Universidade do Porto, Porto, Portugal.

***To whom correspondence should be addressed:**

Renato Socodato (renato.socodato@ibmc.up.pt) and Roberto Paes-de-Carvalho (robpaes@vm.uff.br)

Supplementary Materials

Figure. S1.

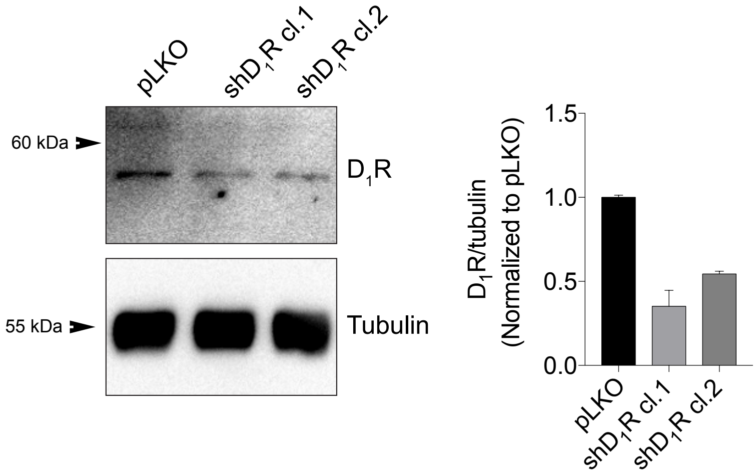

Fig. S1. Knockdown of D_1R in retinal neuronal cultures.

Representative Western blot for D_1R in extracts from retinal cultures expressing the empty vector pLKO or D_1R shRNA (clone 1 or clone 2). Tubulin was used as the total. N = 2 different and independent cultures, One-way ANOVA.

Figure S2.

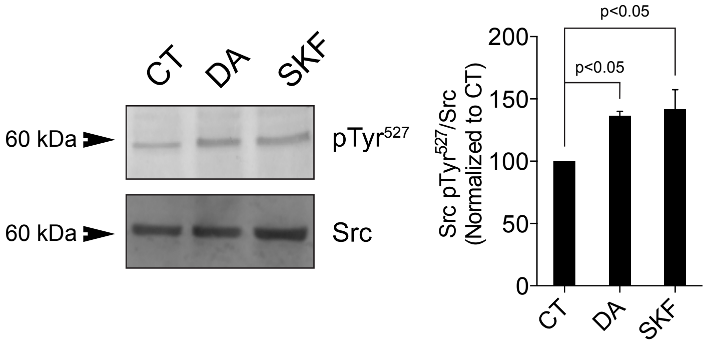

Fig. S2. Src phosphorylation in the intact retina.

Intact retinal tissue (embryonic day 11) was acutely isolated in ice-cold CMF and incubated with DA (50 μM) or SKF-38393 (10 μM) for additional 30 min. Western blotting on tissue lysates evaluated Src pTyr^{527} and Src levels. Data are the mean ± SEM. N = 3 different retinas, One-way ANOVA.

Figure S3.

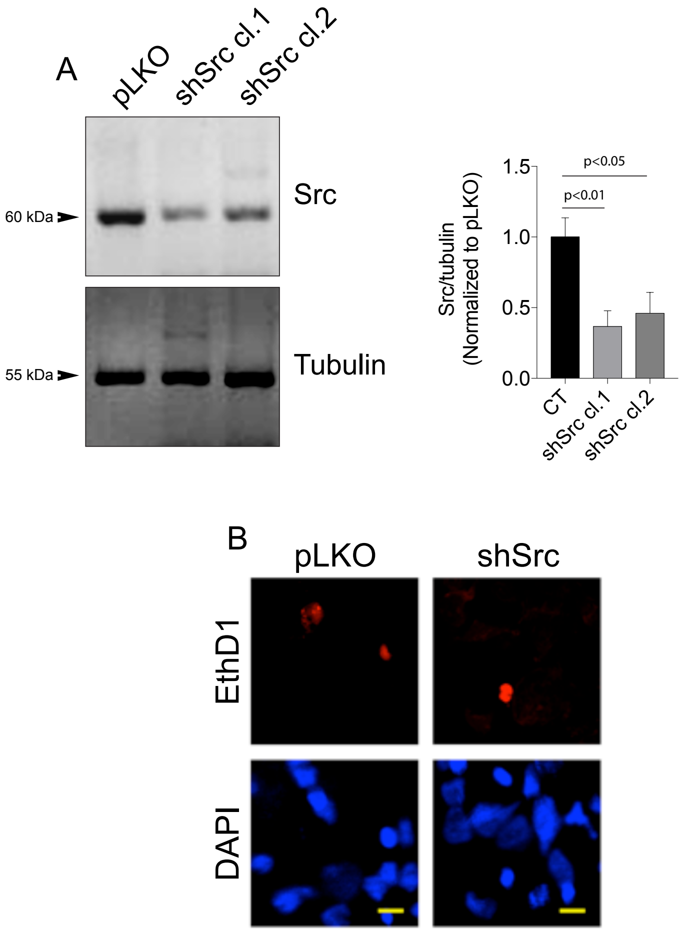

Fig. S3. Knockdown of Src in retinal neuronal cultures.

A: Representative Western blot for Src in extracts from retinal neuronal cultures expressing the empty vector pLKO or Src shRNA (clone 1 or clone 2). Tubulin was used as the total. N = 3-4 different and independent cultures, One-way ANOVA.

B: Src knockdown does not affect cell viability. Representative images of EthD1 labeling (marker for cell death; red) in retinal neuronal cultures expressing the empty vector pLKO or Src shRNA. N = 3 different and independent cultures. Calibration bar = 10 μm.

Figure S4.

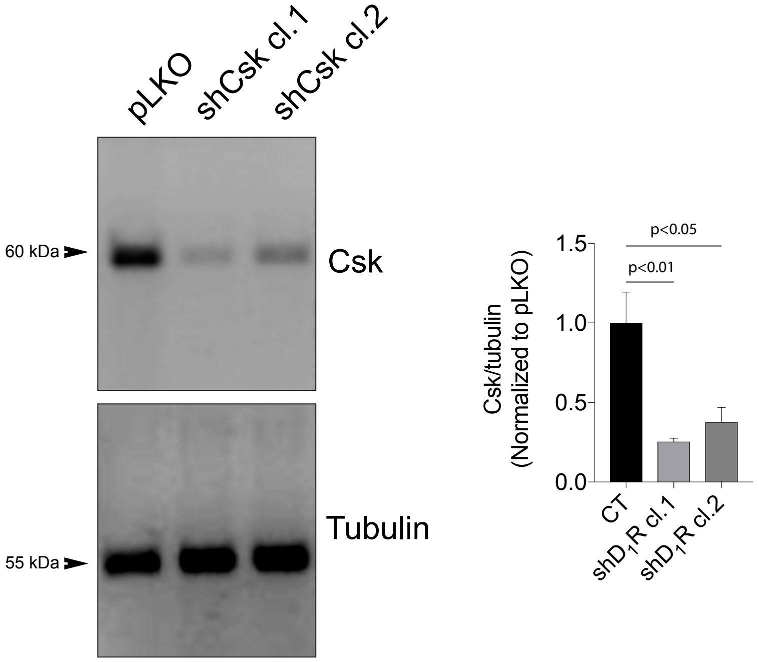

Fig. S4. Knockdown of Csk in retinal neuronal cultures.

Representative Western blot for Csk in extracts from retinal neuronal cultures expressing the empty vector pLKO or Csk shRNA (clone 1 or clone 2). Tubulin was used as the total. N = 3 different and independent cultures, One-way ANOVA.

.

Figure S5.

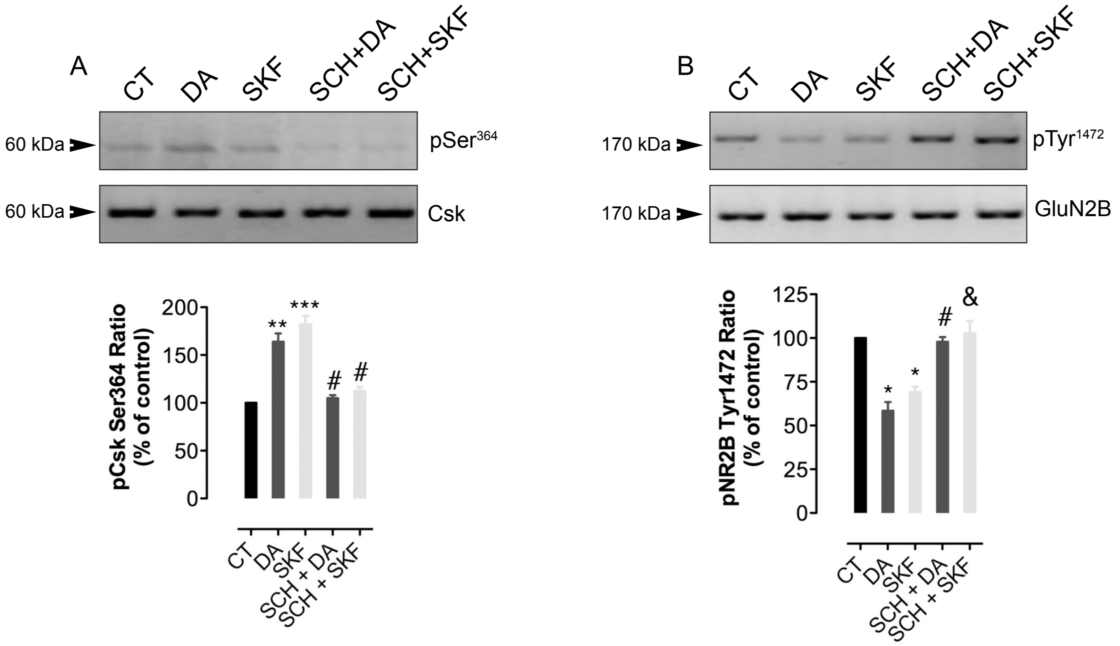

Fig. S5. Csk and GluN2B phosphorylation in the intact retina.

A and B: Intact retinal tissue (embryonic day 11) was acutely isolated in ice-cold CMF and pre-incubated with SCH (50 μM; 10 min) and then incubated with DA (50 μM) or SKF-38393 (10 μM) for additional 30 min. Western blotting on tissue lysates evaluated Csk pSer^{364} and Csk levels (**A**) or GluN2B pTyr^{1472} and GluN2B levels (**B**). *p<0.05, **p<0.01, ***p<0.001 in relation to CTs; #p<0.05, in relation to DA (DA in **A-B**) or in relation to SKF-38393 in **B**; & p<0.05 in relation to SKF-38393 in GluN2B pTyr^{1472} in **A**. Significance was determined by One-way ANOVA, N = 3-4 different retinas.

Figure S6.

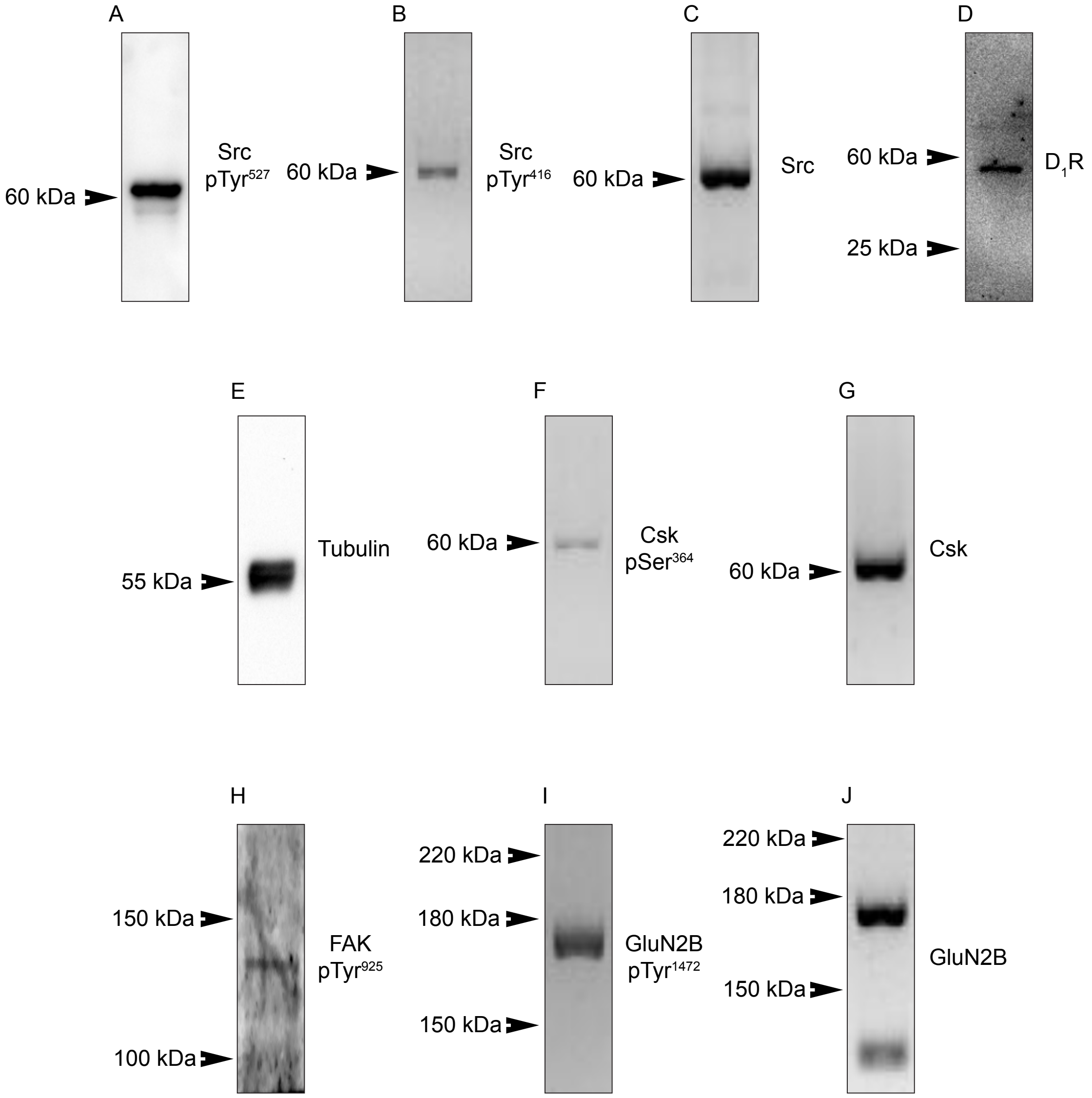

Fig. S6. Validation of antibodies used in retinal neuronal cultures by Western blotting. Retinal neuronal cultures were lysed and cell extracts were prepared for Western blot using different antibodies. Gels are representative of 2-3 different and independent cultures.

Fig. S7. Uncropped Western blots related to main Fig.1.

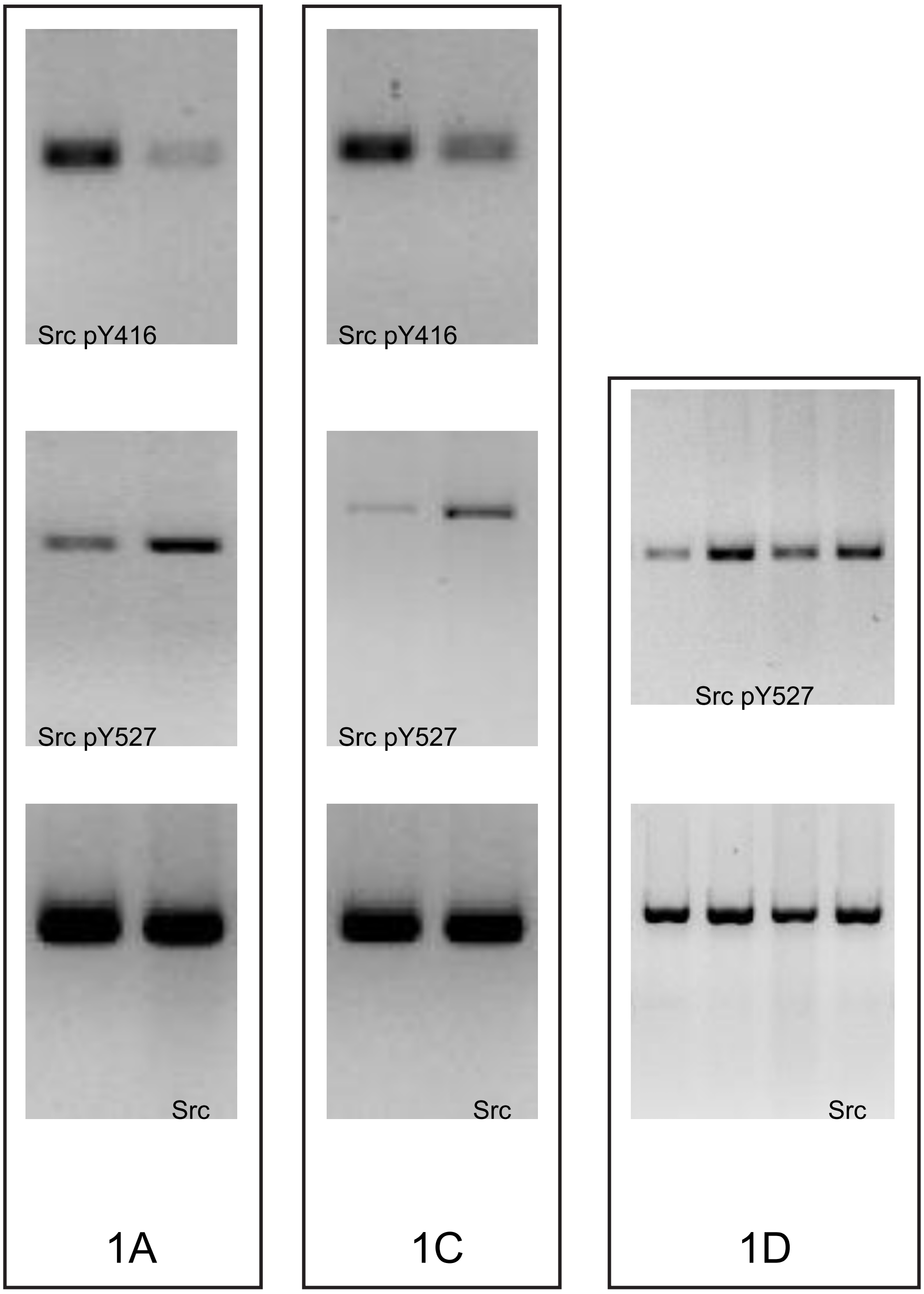

Fig. S8. Uncropped Western blots related to main Fig.2.

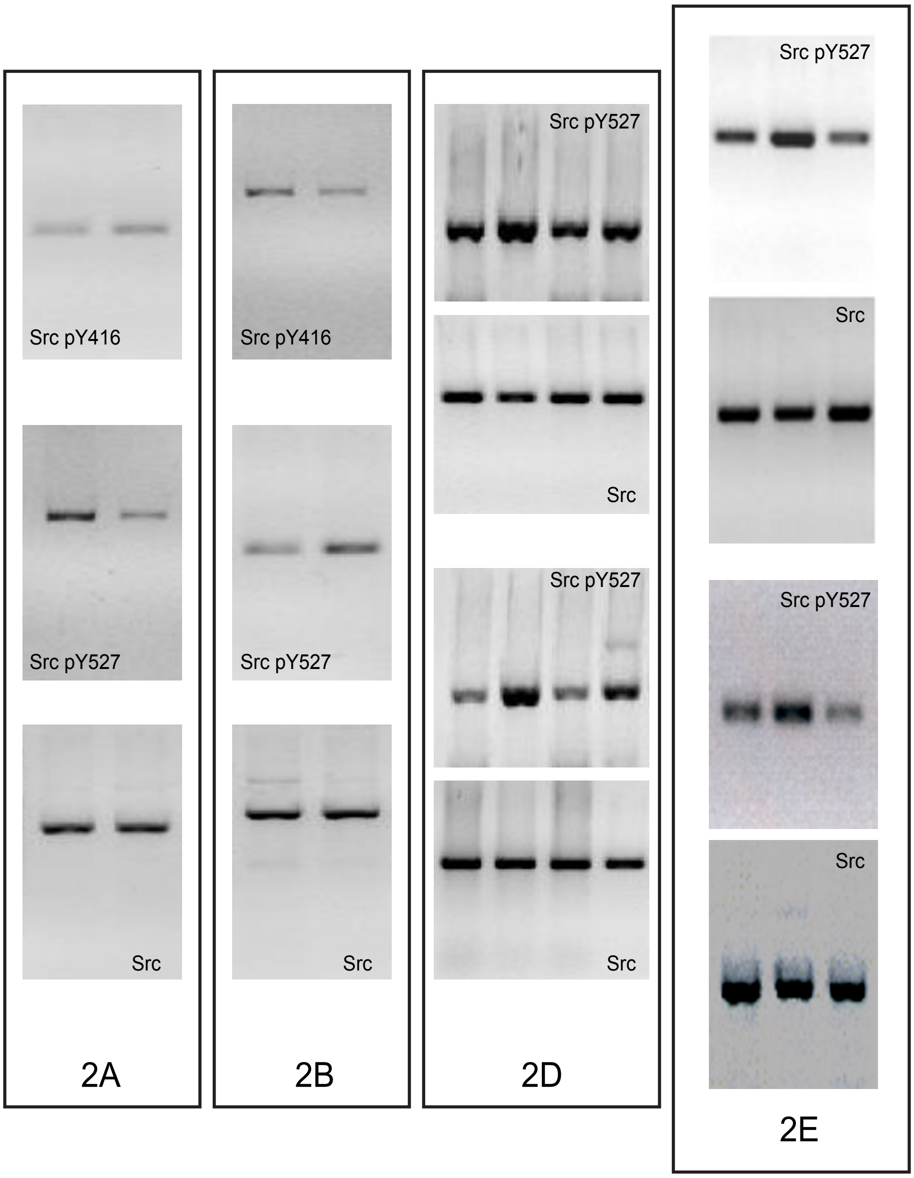

Fig. S9. Uncropped Western blots related to main Fig.3.

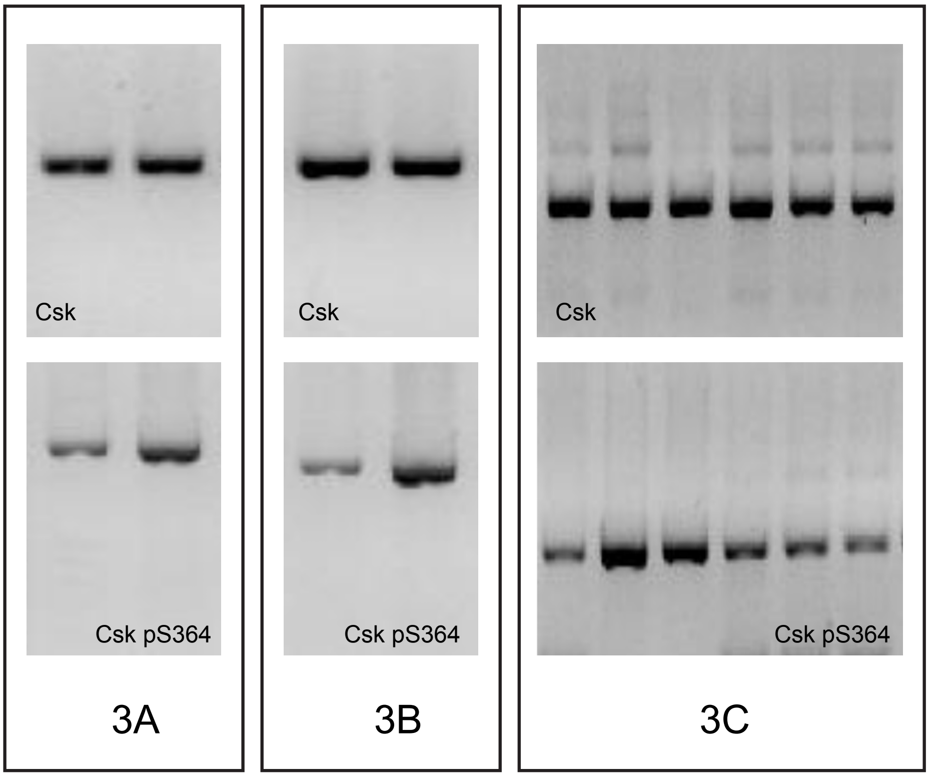

Fig. S10. Uncropped Western blots related to main Fig.4.

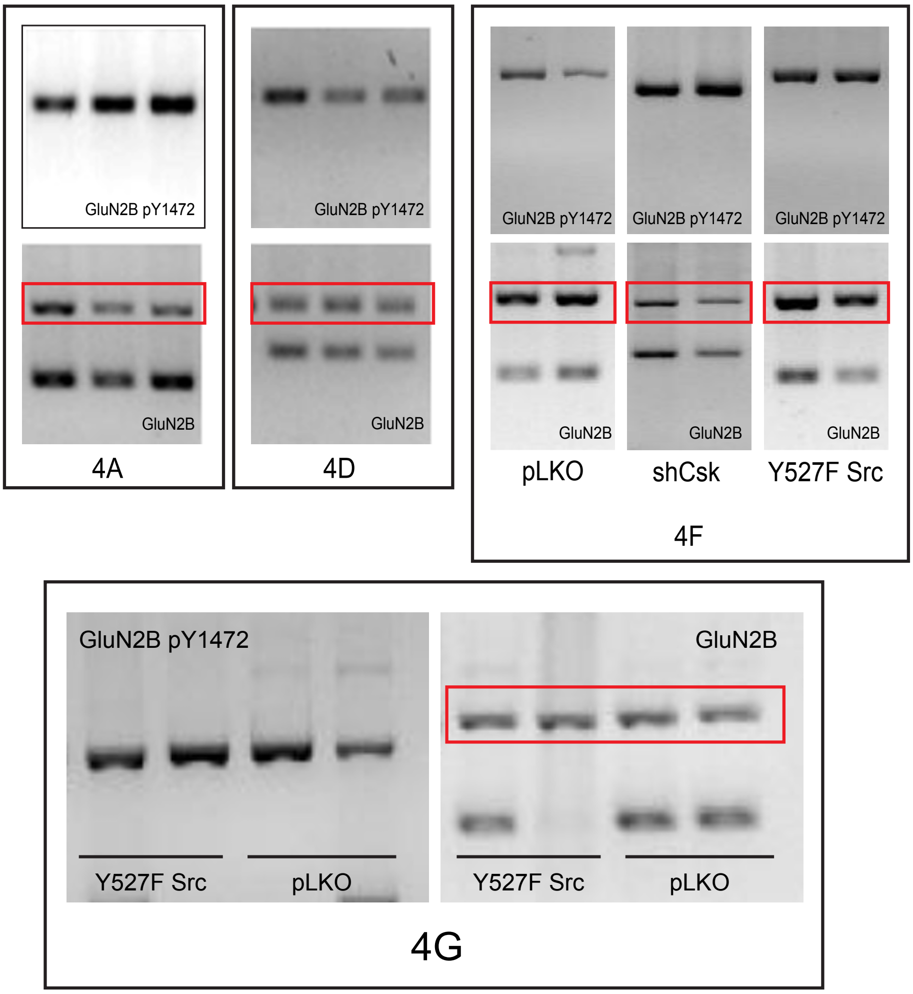

Supplement: Supplementary Figures [file srep40912-s1.pdf]
